# Functional Translatome Proteomics Reveal Converging and Dose-Dependent Regulation by mTORC1 and eIF2α

**DOI:** 10.1016/j.molcel.2019.11.010

**Published:** 2020-02-20

**Authors:** Kevin Klann, Georg Tascher, Christian Münch

**Affiliations:** 1 Institute of Biochemistry II, Faculty of Medicine, Goethe University, Frankfurt am Main, Germany; 2 Frankfurt Cancer Institute, Frankfurt am Main, Germany; 3 Cardio-Pulmonary Institute, Frankfurt am Main, Germany

**Keywords:** proteomics, integrated stress response, translation, stress response, mTOR, unfolded protein response, cap-dependent translation, SILAC, TMT, pulse labeling

## Abstract

Regulation of translation is essential during stress. However, the precise sets of proteins regulated by the key translational stress responses—the integrated stress response (ISR) and mTORC1—remain elusive. We developed multiplexed enhanced protein dynamics (mePROD) proteomics, adding signal amplification to dynamic-SILAC and multiplexing, to enable measuring acute changes in protein synthesis. Treating cells with ISR/mTORC1-modulating stressors, we showed extensive translatome modulation with ∼20% of proteins synthesized at highly reduced rates. Comparing translation-deficient sub-proteomes revealed an extensive overlap demonstrating that target specificity is achieved on protein level and not by pathway activation. Titrating cap-dependent translation inhibition confirmed that synthesis of individual proteins is controlled by intrinsic properties responding to global translation attenuation. This study reports a highly sensitive method to measure relative translation at the nascent chain level and provides insight into how the ISR and mTORC1, two key cellular pathways, regulate the translatome to guide cellular survival upon stress.

## Highlights

• A proteomics method quantifies nascent protein chains minutes after labeling
• The integrated stress response and mTORC1 share translation targets
• Individual protein translation levels are driven by global translation status

## Graphical Abstract

**Figure:**
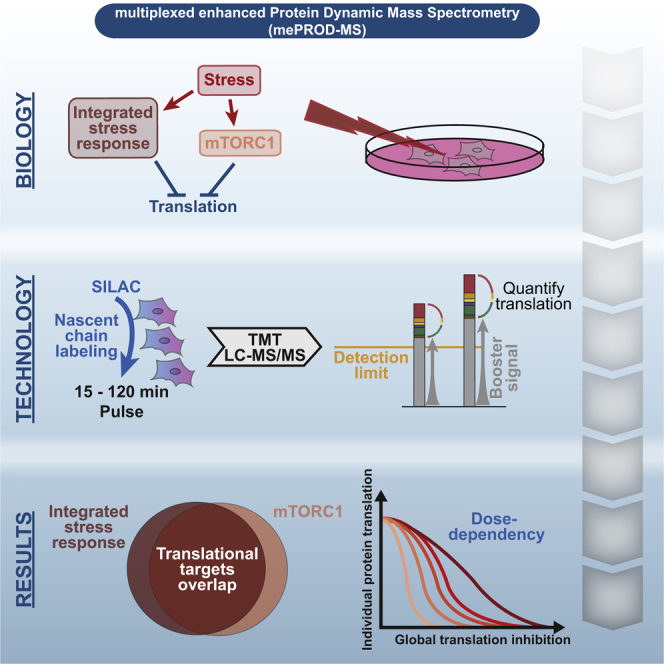

## Teaser

Klann et al. developed a proteomics method that uses signal amplification to enable acute translation measurements. Employing this method, they reveal that the integrated stress response and mTORC1 inhibit translation of the same set of proteins. Target specificity is defined by global translation attenuation in a dose-dependent manner.

## Introduction

Stress response mechanisms control cellular fate through multi-layered regulation. Attenuation of translation is a rapid cellular response triggered by various stresses, such as the induction of the integrated stress response (ISR) and mTOR inhibition (Sonenberg and Hinnebusch, 2009). The ISR is driven by the phosphorylation of eukaryotic initiation factor 2 subunit 1 (eIF2α/EIF2S1) by one of four eIF2α kinases (EIF2ΑK1-4) and activated by diverse stresses, such as heme depletion (EIF2ΑK1/HRI), viral infection (EIF2ΑK2/PKR), ER stress (EIF2K3/PERK), or amino acid deprivation (EIF2ΑK4/GCN2) (Taniuchi et al., 2016). Phosphorylation of eIF2α causes tightened binding to guanine nucleotide exchange factor eIF2B, preventing formation of the 40S preinitiation complex and leading to cellular translation attenuation (Kozak, 1999, Krishnamoorthy et al., 2001). Control of cellular translation by the ISR plays a central role in various diseases, such as diabetes, cancer, and viral infection (Back et al., 2009, Clavarino et al., 2012, Pakos‐Zebrucka et al., 2016).

The mammalian target of rapamycin complex 1 (mTORC1) is the second major pathway mediating translational control in cells. Under basal conditions, mTORC1 phosphorylates the EIF4E binding proteins EIF4EBP1-3 and ribosomal protein S6 kinase (p70S6K1) (Sonenberg and Hinnebusch, 2009). EIF4EBP phosphorylation leads to dissociation from eIF4E, enabling binding to eIF4G and the formation of the initiation complex at the 5′-cap of mRNAs. Phosphorylation of p70S6K1 activates its kinase function and regulates translation by targeting EEF2K, EIF4B, and ribosomal protein S6 (Holz et al., 2005, Raught et al., 2004, Wang et al., 2001). In response to low nutrient concentrations, mTORC1 becomes inactivated, resulting in hypo-phosphorylated EIF4EBP that subsequently binds eIF4E and represses cap-dependent translation. Consequently, mTORC1 has major control over cellular behavior, and its regulation is modulated in numerous cancers (Sabatini, 2006). Studies monitoring EIF4EBP- and eIF4E-dependent translation regulation identified a small subset of mRNAs to be controlled via this route (De Benedetti and Graff, 2004, Colina et al., 2008, Dowling et al., 2010, Graff and Zimmer, 2003, Roux and Topisirovic, 2012).

Despite both pathways regulating (albeit different) processes in translation initiation, eIF2α and mTORC1 are generally viewed as separate, translation-controlling pathways with specific outcomes (Wengrod and Gardner, 2015). A major focus of study of these translation-regulating pathways has been the analysis of downstream effects. Global analyses identifying and quantifying the specific translational output of translation regulation by eIF2α and mTOR have largely been carried out by ribosome profiling (Hsieh et al., 2012, Jiang et al., 2017, Reid et al., 2014, Sidrauski et al., 2015, Thoreen et al., 2012). These studies revealed a low number of differentially translated transcripts, despite showing extensive global downregulation of translation. This is largely due to a normalization procedure bias (Chen et al., 2015, McGlincy and Ingolia, 2017) that redistributes translation values back to unchanged global relative translation rates. As a result, transcripts do not reach sufficient statistical significance and/or fold changes (FC) to be identified as downregulated during translation attenuation by eIF2α or mTORC1 (Masvidal et al., 2017). Until today, conclusive datasets representing the set of proteins with reduced translation following eIF2α- or mTORC1-driven translation attenuation are not available. Thus, it remains unclear which proteins are translationally regulated by eIF2α and mTORC1 and whether these sets are indeed distinct and may be discriminated by additional features besides being capped.

In the last years, mass spectrometry (MS) approaches have helped in assessing protein dynamics by detecting protein degradation and synthesis, employing pulse-labeling of nascent peptide chains with heavy amino acid isotopes (SILAC) or click-reactive amino acids/puromycin (Becher et al., 2018, Jovanovic et al., 2015, Mathieson et al., 2018, Savitski et al., 2018, Schwanhäusser et al., 2009, Welle et al., 2016). A major limiting factor in the use of pulse-labeling newly synthesized proteins is the low stoichiometry of labeled proteins, preventing accurate, precise, and in-depth quantification (Münch and Harper, 2016). For basal protein degradation and synthesis experiments that monitor proteins over several days (Schwanhäusser et al., 2011), this issue has been overcome by combining pulse-labeling MS and tandem-mass tag (TMT)-based multiplexing (Welle et al., 2016). TMT allows isobaric tagging and pooling of up to 11 samples into one multiplexed sample. Combining pulse-labeling with TMT can achieve a balanced distribution of unlabeled and labeled protein species. However, these methods do not allow studying acute processes in response to cellular stimulation, such as cellular stress affecting translation via eIF2α/mTORC1.

Here, we describe multiplexed enhanced protein dynamics (mePROD) MS that allows quantifying heavy label incorporation after very short labeling times without a loss of depth or accuracy. mePROD is based on addition of a booster channel that increases the signal of interest in a TMT-multiplexed and dynamic SILAC-labeled sample. This method enables acute monitoring of global translation rates and captures global translation attenuation by quantifying newly synthesized proteins. Employing mePROD, we provide insight into the global rearrangement of cellular translation upon modulation of eIF2α and/or mTORC1 activity to reveal common mechanisms in these distinct stress responses.

## Results

### mePROD Enables Detecting SILAC Incorporation at Low Stoichiometry

**Figure 1:**
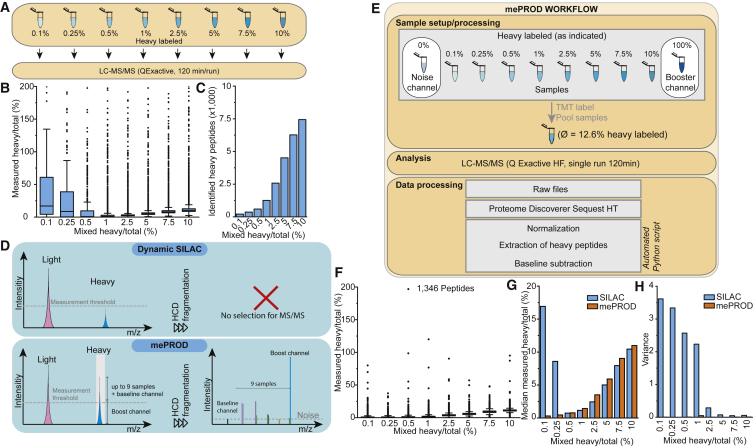
mePROD Proteomics Overcomes Low Accuracy and Identifications of Peptides at Low Heavy-to-Light Ratio (A) Scheme of experimental design. Heavy and light peptides were mixed at indicated ratios. (B) Measured heavy to total ratios on peptide level. Boxes indicate 25%/50% quartiles and the median; whiskers show standard deviation. (C) Number of heavy labeled peptides quantified in (B). (D) Underlying principle of mePROD to increase signals of interest. Low labeling stoichiometry prevents reaching the measurement threshold using standard dynamic SILAC approaches (top). In mePROD, a booster channel comprised of a fully heavy labeled proteome boosts the signal of interest above the MS^1^ detection level (bottom). Heavy/total ratios for individual samples are then then determined from TMT signals quantified in MS^2^ (right). (E) Experimental mePROD design and data processing. Samples from (A) were combined with noise and booster channels, TMT-labeled, pooled, analyzed by LC-MS^2^, and raw files processed. Reporter ion intensities for peptides were sum normalized and heavy peptide intensities extracted. To enhance accuracy, baseline values derived from the non-SILAC labeled channel were subtracted from each peptide. (F) Samples as in (A) were analyzed using mePROD (using 1/8^th^ of the LC-MS^2^ machine time used in A). Comparison of measured versus expected heavy/total ratios. Boxes indicate 25%/50% quartiles and the median; whiskers show standard deviation. (G and H) Comparison of median measured heavy/total peptide ratios (G) or variance (H) for samples measured by SILAC or mePROD. See also Figure S1.

Effects of stress on cellular translation are rapid and occur within a few hours (Prostko et al., 1993). Therefore, time-resolved methods are required to quantify translatome changes upon acute stresses. However, since the median half-life of proteins is about 46 h (Schwanhäusser et al., 2011), only a small fraction of every protein is to be newly synthesized in the first hours upon cellular modulation. To simulate this situation, we mixed heavy and light peptides at set ratios to assess the capability of pulsed-SILAC to monitor acute changes in translation. Peptides derived from digested HeLa whole-cell lysates grown in light SILAC medium (from here on referred to as light) or in heavy SILAC medium (K8 and Arg10 labeled, from here on referred to as heavy) were mixed at ratios ranging from 0.1% to 10% heavy/total (H/T) and analyzed by LC-MS^2^ (Figure 1A). Examining the range of measured H/T ratios revealed low accuracy, in particular for low H/T ratios, with the measured median for samples mixed at an H/T ratio of 0.1% deviating by about 100-fold from the expected ratio (Figure 1B). At low H/T ratios, only 216 peptides were identified, at least partially explaining the high variation observed (Figure 1C). The number of identified peptides increased at higher H/T ratios consistent with an inherent H/T threshold required for correct quantification of H/T ratios. Thus, as previously described (Schwanhäusser et al., 2011), pulsed SILAC allowed us to monitor relative translation rates; however, for low H/T ratios, representing translation activity occurring in the time span of few hours, identification rates and accuracy of quantification was insufficient, since heavy peaks were below the detection limit (Figure 1D, top).

When combining pulsed-SILAC with TMT-labeling, the MS^1^ signals of (heavy) peptides sum up across all samples due to the isobaric nature of the TMT tag. We hypothesized that we could take advantage of that property by adding a booster channel containing peptides from fully SILAC-labeled cell lysates. This approach can increase the summed heavy peak intensity across all samples and enable accurate measurement of protein translation at small H/T ratios (Figure 1D, bottom). To validate this hypothesis, we prepared a TMT-labeled 10-plex sample containing equimolar amounts of a dilution range of H/T ratios (from 0.1%–10% H/T, as in Figure 1A), a fully SILAC-labeled cell digest to increase the signal of heavy-labeled MS^1^ peaks (booster channel), and a non-SILAC-labeled cell digest (noise channel) to determine noise levels and allow baseline subtraction for individual peptides (Figure 1E). We identified and quantified 1,346 heavy peptides for all channels, improving the identification rate by up to 6-fold across the range of measured H/T ratios (Figure 1F) while using 12.5% of the machine time necessary for individual SILAC samples (Figures 1A and 1B). mePROD correctly determined H/T ratios across the whole range (Figure 1G) and improved accuracy by three orders of magnitude, especially for lower H/T ratios (Figure 1H). Together, these results demonstrated the capacity of mePROD to both increase the identification rate of H/T ratios and accuracy.

### Measuring Translation by mePROD

**Figure 2:**
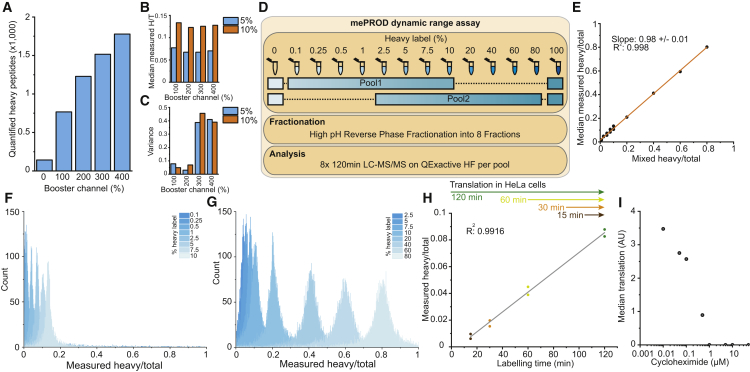
High Dynamic Range of mePROD to Measure Heavy/Light Peptide Ratios and Translation (A–C) mePROD 6-plex samples were prepared mixing noise channel, two replicates of each 5% and 10% heavy/total peptide mix, and indicated amounts (relative to samples) of fully labeled booster channel. Shown are numbers of identified and quantified peptides (A), measured heavy/total (H/T) ratios (B), and variance (C). (D–G) Experimental design (D). Two mePROD 10-plex samples including samples ranging from 0.1% to 10% and 2.5% to 80% heavy labeled peptides were mixed with noise and booster channel as indicated, fractionated, and analyzed. Comparison of measured versus expected heavy/total ratios (E). Histograms depicting count distributions of measured heavy/total ratios of 10-plexes ranging from 0.1%–10% (F) and 2.5%–80% (G). (H) Measured heavy/total peptide ratios of cells incorporating heavy amino acids into newly synthesized proteins for different lengths of time measured by mePROD (n = 2). (I) Cells were pre-treated for 2 h with indicated concentrations of cycloheximide and pulse-labeled for an additional 2 h with SILAC medium. Median global translation was measured and plotted against cycloheximide concentration. See also Figure S1.

We next tested whether increasing the amount of booster channel added could further improve identification rates. Indeed, increasing the amount of booster channel resulted in higher identification rates of heavy SILAC-labeled peptides without affecting overall quantification results (Figures 2A and 2B). As the variance of quantification increased 4- to 5-fold with booster channel levels at or above 300% (Figure 2C), we continued with using the booster channel at double-molar ratio (200%).

Next, we analyzed the dynamic range and accuracy of mePROD (Figure 2D). Plotting measured against input ratios showed linear behavior across the whole range with a R^2^ value of 0.998 (Figures 2E–2G), demonstrating the capability of mePROD to accurately measure a wide dynamic range of H/T peptide ratios. Comparing MS^2^ versus MS^3^ methods did not reveal any major changes (Figure S1A), with the addition of the baseline channel being sufficient to overcome ratio compression (Figure S1B).

To determine the applicability and temporal resolution of mePROD in cells, we labeled HeLa cells for 15–120 min and measured H/T ratios (Figure 2H), revealing linear behavior (R^2^ of 0.9916) and indicating that 15 min of labeling time was sufficient for quantification (Figures 2F–2H, S1C, and S1D). To determine the dynamic range of mePROD for translation rate analysis, we inhibited total cellular translation by addition of different concentrations of cycloheximide (CHX) and analyzed the global translation levels. We observed a CHX concentration dependent decrease in global translation across the full range (Figure 2I). Together, these findings demonstrated that mePROD could determine acute changes in cellular translation with high accuracy.

### mePROD MS Quantifies the Functional Translatome upon UPR Induction

**Figure 3:**
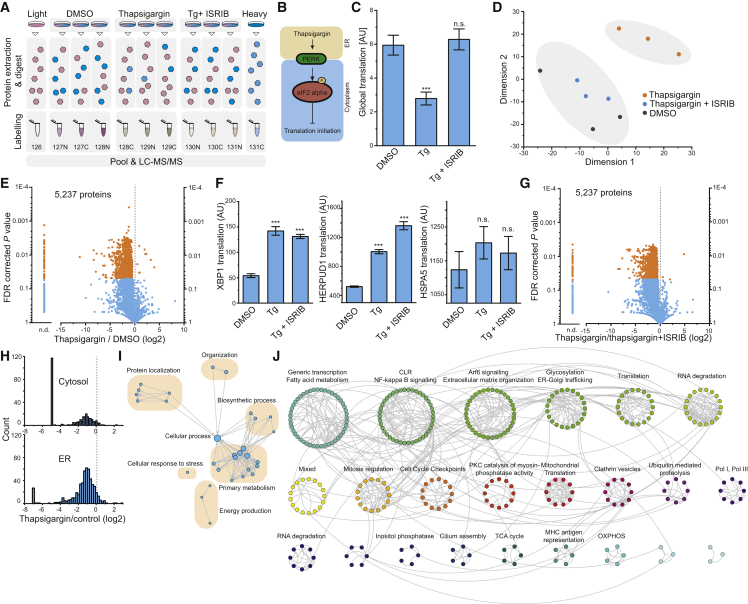
Changes in the Cellular Translatome upon Activation of the Integrated Stress Response by Protein Misfolding in the Endoplasmic Reticulum (A) Experimental layout. Three different conditions were pooled (in triplicate) with noise and booster channels and analyzed by mePROD MS. (B) Scheme of translational repression during the UPR, induced by PERK activation. (C and D) Global translation levels assessed by mePROD MS for cell treated with DMSO, 1 μM thapsigargin (Tg), or 1 μM thapsigargin and 500 nM ISRIB (Tg + ISRIB) for 2.5 h. Shown are median intensities of heavy labeled peptides (C). Error bars indicate standard deviation (n = 3). ^∗∗∗^p < 0.001; n.s., not significant (two-sided, unpaired Student’s t test with equal variance). AU, arbitrary units. Multidimensional scaling analysis of samples standardized by unit variance (D). (E) Volcano plot showing fold change of relative translation versus adjusted p value of thapsigargin versus control treated cells. Orange dots indicate significantly changing proteins (p values < 0.05 and fold change [log2] ≤ −0.5 or ≥ 0.5). Samples for which abundances in thapsigargin treated samples dropped below baseline and no fold change could be calculated are indicated as not determinable (n.d.). (F) Changes in translation levels of XBP1, HERPUD1, and HSPA5 (better known as BIP) measured by mePROD MS. Mean heavy abundance was plotted with error bars indicating standard deviation (n = 3). ^∗∗∗^p < 0.001; n.s., not significant (two-sided, unpaired Student’s t test with equal variance). Tg, thapsigargin. (G) Volcano plot showing fold change versus adjusted p value between thapsigargin and thapsigargin+ISRIB treated samples. Significantly changing proteins in orange (as in E). (H) Histogram depicting translation changes for cytosolic versus endoplasmic reticulum resident proteins. (I) EnrichmentMap network showing significantly (q value < 0.001) enriched GO terms for proteins without significantly changed relative translation rates upon thapsigargin treatment. (J) ReactomeFI cluster analysis for proteins not changing relative translation rates upon thapsigargin treatment. Proteins were FI annotated, clustered, and clusters analyzed for significantly enriched Reactome pathways (q value < 0.001). The most prominent pathway of each cluster is indicated. Connecting lines show interaction of protein nodes. See also Table S1 and Figures S2–S4.

Although the unfolded protein response (UPR) causes severe ablation of global translation via phosphorylation of eIF2α (Harding et al., 2000), the precise set of individual proteins, whose translation is reduced upon UPR induction, remains unknown. We determined if mePROD can measure acute changes in translation and identify global translation effects that faithfully reproduce the ∼50% ablation of translation observed by ^35^S incorporation experiments (DuRose et al., 2009). Cells were treated in triplicate with DMSO, 1 μM thapsigargin, or a co-treatment of 1 μM thapsigargin and 500 nM ISRIB (a small molecule reversing the effect of eIF2α phosphorylation (Sidrauski et al., 2015)) and translation measured after 2 h of label incorporation (Figures 3A and 3B; Table S1). Global translation attenuated by approximately 50%, confirming data observed by other methods (Preston and Hendershot, 2013), and was fully reversed by ISRIB (Figure 3C). In addition to detecting global changes in translation, we quantified individual relative translation levels of 5,237 proteins. Multidimensional scaling analysis (MDS) showed replicates clustering together and that samples co-treated with thapsigargin and ISRIB behaved like control samples (Figures 3D and S2). We next investigated proteins displaying significant changes in translation upon UPR induction (adjusted [adj.] p value < 0.05, FC (log2) < 0.5 or > 0.5), when compared to control treatment. Translation of 1,780 proteins was significantly decreased and nine proteins showed increased translation upon UPR induction (Figure 3E). Proteins with increased translation upon UPR contained known UPR targets, such as XBP1 and HERPUD1, that are mediated by the UPR receptor IRE1 and are thus not reliant on eIF2α and not affected by ISRIB (Lee et al., 2003, Miura et al., 2010, Ron and Walter, 2007, Yoshida et al., 2001) (Figure 3F). Taken together, mePROD can measure acute changes in translation with high overall depth. Strikingly, mePROD translation data strongly overlapped with data derived from ribosome profiling under similar conditions while revealing a much more significant portion of proteins reduced upon UPR induction (Paolini et al., 2018, Reid et al., 2014, Sidrauski et al., 2015) (Figure S3). Moreover, an extensive rearrangement of the cellular translatome upon UPR induction was driven by eIF2α, as shown by the nearly complete reversal of translational attenuation when co-treating with ISRIB (Figures 3C, 3D, 3G, and S4A–S4C). Notably, there was no apparent difference in overall translation ablation of cytosolic versus ER-resident proteins (Figure 3H).

We next sought to analyze the fraction of 623 proteins whose translation did not change upon thapsigargin treatment, suggesting that translation of their mRNAs is resistant to the eIF2α phosphorylation-induced changes observed. GO term enrichment analysis of biological processes showed six significant clusters (Figure 3I). However, the identified clusters overlapped with clusters found for proteins with decreased translation (Figure S4D). This observation strongly suggested that global GO analyses could not explain the observed complexity as subsets of the generalized GO terms appear to be regulated in different ways. Therefore, we analyzed the set of proteins with unchanged translation after thapsigargin treatment on the level of individual proteins using ReactomeFI gene set analysis (Figure 3J). We found 23 different clusters of interacting proteins, with a cluster size larger than two, annotated to different cellular pathways (q < 0.001). The identity of those clusters suggests that stress conditions in the ER attenuate global translation while maintaining critical parts of central pathways to maintain cell function. In summary, we could employ mePROD MS to precisely and accurately measure protein translation at high sensitivity (i.e., below 2 h) to determine the effect of acute thapsigargin treatment on translation.

### Different Stress Response Pathways Share Common Translational Programs

**Figure 4:**
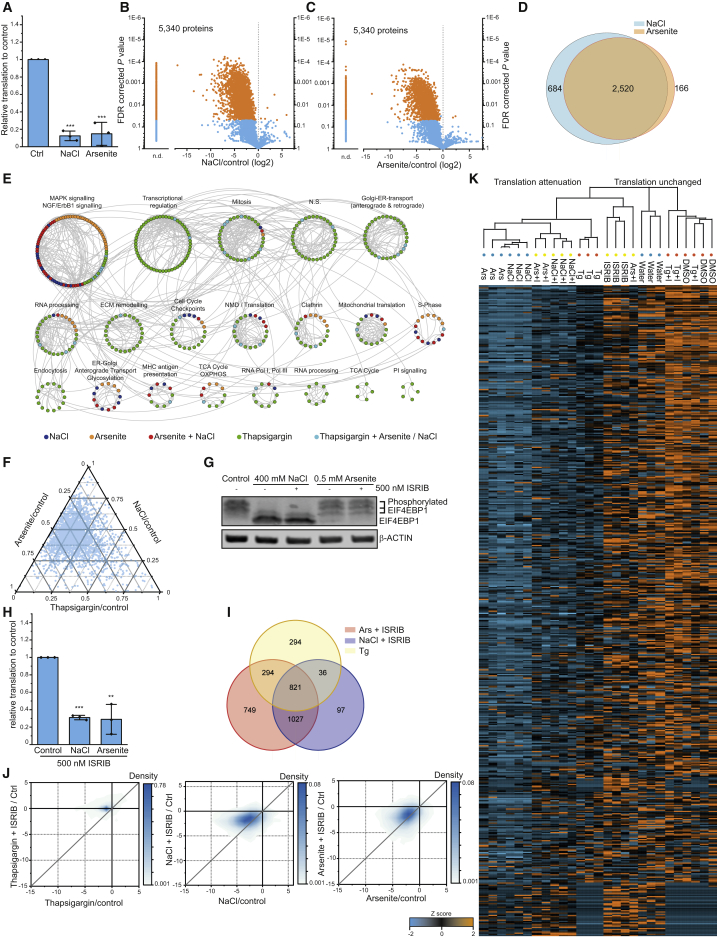
Translatome Repression Patterns Shared across Stress Response Pathways (A) Mean median translation levels of samples treated with water, 400 mM NaCl, or 0.5 mM arsenite for 2.5 h measured by mePROD MS. Individual values are indicated. Error bars show standard deviation (n = 3). ^∗∗∗^p < 0.001 (Two-way Student’s t test). (B and C) Volcano plot showing fold change versus p value for NaCl (B) or arsenite (C) versus control. Orange dots indicate significantly changing proteins. n.d., not determinable (intensities for treated samples below noise levels). (D) Overlap between translational repressed proteins (fold change [log2] < −0.5 and adj. p < 0.05) in NaCl or arsenite-treated cells. (E) ReactomeFI cluster network (q value < 0.001). Unchanged proteins in three treatments (thapsigargin, NaCl, arsenite, fold change [log2] > −0.35) were merged into one network, clustered by functional enrichment, and clusters analyzed for reactome pathway enrichment. Proteins were colored according to dataset and most prominent pathways of each cluster annotated. Connecting lines show interaction of protein nodes. (F) Ternary plot comparing fold changes for each protein between thapsigargin, NaCl, or arsenite treatments. For each protein and treatment, fold changes were summed and ratios to total fold changes determined and plotted. (G) Western blot showing phosphorylation of EIF4EBP1 upon control, NaCl, or arsenite treatment with or without ISRIB co-treatment. EIF4EBP1 antibody reveals both non-phosphorylated and phosphorylated species. (H) Cells were treated as in (A) with addition of 500 nM ISRIB. Histogram of global translation relative to control with standard deviation (n = 3). ^∗∗^p < 0.01; ^∗∗∗^p < 0.001 (Two-sided Student’s t test). (I) Overlap of proteins translationally repressed via eIF2α phosphorylation (by thapsigargin) and proteins not showing reversal by co-treatment with ISRIB and NaCl and arsenite. (J) Density plots showing translation fold changes for each protein between stressor alone and co-treatment with ISRIB. Grey lines represent the reference line for equal fold changes. (K) Heatmap and hierarchical clustering summarizing result for all shown treatments (Figures 3 and 4). Datasets were combined, Z scores calculated, and hierarchical clustering performed using Euclidean distance between the samples. Depicted are Z score values for each treatment and replicate (n = 3). Colored circles indicate the 11plex experiment in which the sample was included. I, ISRIB; Ars, arsenite; Tg, thapsigargin. See also Tables S2 and S3 and Figures S4 and S5.

We next asked if diverse ISR activating stressors reshape the cellular translatome in a similar fashion. Therefore, we induced ISR-dependent eIF2α phosphorylation with commonly used treatment paradigms for osmotic or oxidative stress (400 mM sodium chloride or 500 μM arsenite, respectively) (Andreev et al., 2015, Rabouw et al., 2019, Taniuchi et al., 2016) and monitored translation (Figure 4A; Table S2). Consistent with previous studies (Bevilacqua et al., 2010, McEwen et al., 2005), both treatments induced extensive translational attenuation (Figure 4A). Quantifying proteins on an individual level showed 3,204 proteins and 2,686 proteins with significant translation decrease for osmotic stress and oxidative stress, respectively (Figures 4B and 4C), with an overlap of ∼87% (Figure 4D). As expected, only few proteins showed increased translation. When comparing the translational effects of these stresses with the ones induced by the UPR, we found distinctively different classes of clusters for specific treatments (Figure 4E): (1) several clusters were exclusive to ER stress. (2) Clusters shared between all three treatments, suggesting a core requirement to maintain cell function. (3) One cluster for ER-to-Golgi transport only observed upon NaCl or arsenite treatment. Strikingly, this cluster was distinct from another ER-to-Golgi transport cluster that is specific to thapsigargin treatment, revealing that different subsets of this pathway are sensitive to separate stresses (Figure S4E).

### mTORC1 and eIF2α Attenuate Translation of Overlapping Protein Sets

Comparing translatomes for the three ISR-inducing stressors showed an overlap of ∼30% of proteins with reduced translation (Figure S5A). Thapsigargin treatment caused a significantly smaller translational effect on individual proteins than the other treatments, suggesting potential differences in translational control (Figure 4F). Indeed, we found NaCl and arsenite to lead to decreased phosphorylation of the mTORC1 substrate EIF4EBP1 (Figure 4G), consistent with previous publications (Andreev et al., 2015, Plescher et al., 2015). Thus, the observed translatome differences by thapsigargin versus NaCl or arsenite treatments may be driven by mTORC1. To dissect possible overlapping effects of mTORC1 inhibition and eIF2α phosphorylation, we co-treated cells with NaCl or arsenite and ISRIB, which we had found to abolish effects seen by eIF2α phosphorylation (Figure 3). As expected, ISRIB had no effect on mTORC1 activity or eIF2α phosphorylation (Figures 4G and S5B). However, when monitoring global translation using mePROD, translation repression by NaCl or arsenite was partially rescued by ISRIB (Figure 4H; Table S3). We compared the fraction of proteins with rescued translation upon ISRIB, as they should be targeted solely by eIF2α (Figure S5C). Surprisingly, we only found a small overlap in this fraction between all three treatments, while proteins still displaying translation attenuation upon ISRIB and NaCl or arsenite treatment showed a substantial overlap with proteins regulated solely by the ISR/eIF2α (i.e., seen by thapsigargin treatment, Figure 4I). This suggested that eIF2α and mTORC1 might control translation of the same subsets of proteins.

Comparing translation changes of individual proteins following treatment alone or upon co-treatment with ISRIB revealed an increased, but not rescued, translation for the whole population of proteins after ISRIB co-treatment (Figure 4J). The same trend was observed in the global translation behavior (Figures 4A and 4H). Clustering analyses further supported these observations showing similar translation patterns of the co-treatments compared to the single treatments (Figure 4K). ISR and mTORC1 modulation also cause transcriptional changes, such as via modulating ATF4 (Park et al., 2017, Ron and Walter, 2007), that could explain overlapping translation changes across the two pathways. However, when comparing published RNA-seq datasets upon ISR activation or mTORC1 inhibition, we found no changes in global or individual transcript changes that could explain the observed translatome rearrangements (Figures S5D–S5F).

These findings indicate that both translational control pathways—ISR and mTORC1—directly regulate translation of the same proteins. This observation was not apparent from previous Ribo-seq analyses. However, it is consistent with the notion that both ISR and mTORC1 control cap-dependent translation initiation, suggesting that translational targets of the two pathways may indeed overlap. Strikingly, our observations (Figure 4J) also suggested a correlation between individual and global protein translation rates.

### Intrinsic Features Define mTORC1 and ISR Translation Repression Targets

**Figure 5:**
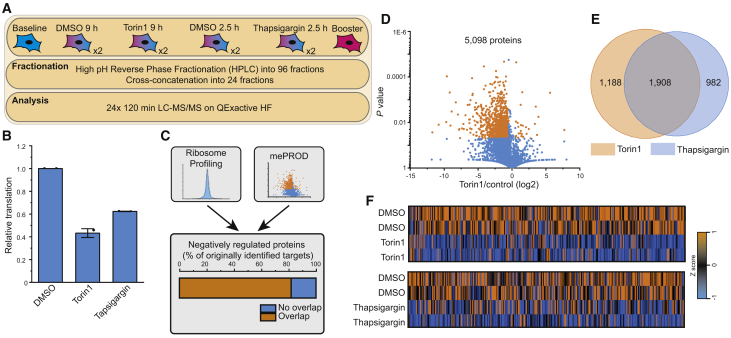
Converging Translatome Regulation by the Integrated Stress Response and mTORC1 (A) Experimental scheme. Cell were treated with thapsigargin or Torin1 for different lengths of time to achieve comparable global translation attenuation. (B) Bar plot showing median global translation levels normalized to the respective control with standard deviation (n = 2). (C) Overlap of proteins with reduced relative translation rates upon Torin1 treatment determined by ribosome profiling data (Thoreen et. al. 2012), or mePROD MS (A). No overlap indicates proteins only showing reduction in ribosome profiling dataset. (D) Volcano plot showing relative translation changes for Torin1 versus control treated cells plotted against p value (n = 2). (E) Venn diagram displaying the overlap of proteins with reduced relative translation (fold change [log2] < −0.5). (F) Heatmap of translation changes for individual treatments and replicates. Data were row-normalized by computing Z scores. See also Table S4.

To further evaluate this hypothesis, we compared translation profiles of cells upon using conditions inhibiting global translation to a similar extent via the ISR (Thapsigargin, 2 h) or mTORC1 (Torin1, 9 h) in one mePROD sample (Figure 5A). Treatment with Torin1 decreased global translation levels by 59% (Figure 5B; Table S4), consistent with previous studies observing ∼65% attenuation (Thoreen et al., 2012). Torin1-induced translatome differences were largely direct effects on translation, not due to transcriptome changes (Figures S5D and S5G), and showed an 87% overlap with previously published Ribo-seq data (Figure 5C) (Thoreen et al., 2012). In addition, mePROD identified over 786 additional, significantly attenuated proteins (Figure 5D). Analyzing the overlap of translationally repressed targets (FC [log2] < −0.5) in both sample sets, we observed 66% of proteins controlled by the ISR and mTORC1 alike (Figures 5E and 5F), confirming a high overlap between translation attenuation targets when inhibiting global translation to similar levels. Overall, comparing changes in the translatome upon treatment with thapsigargin or Torin1 confirmed that (1) the majority of translation targets was indeed regulated by both pathways, and (2) target specificity was not achieved by specific activation of the ISR or mTORC1 inhibition. Thus, translation of sets of proteins did not appear to be controlled by the respective extrinsic pathways (i.e., ISR or mTORC1), instead implying intrinsic factors, such as differential sensitivity of mRNA translation to stress, to control individual protein translation. Consistently, we observed translation of some proteins to be more sensitive to global translation attenuation than others, suggesting inherent differences.

### Individual Protein Synthesis Levels Correlate with Global Translation Rates under Stress Conditions

**Figure 6:**
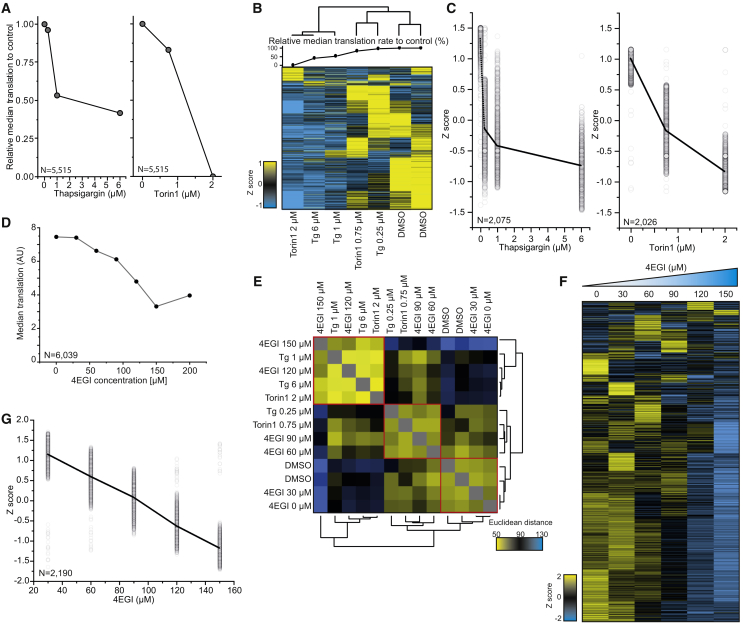
Reduction of Individual Protein Translation Rates Is Defined by the Extent of Global Translation Attenuation (A) Median relative translation for cells treated with DMSO, 0.25 μM, 1 μM, or 6 μM thapsigargin (Tg) for 2.5 h (left panel) or DMSO, 0.75 μM, or 2 μM Torin1 for 9 h (right panel). (B) Heatmap showing Z scores of relative translation rates for individual proteins across treatments (Z scores were calculated for each experiment). Clustering of samples were performed with Euclidean distance. Relative median translation rates compared to control are plotted on top of the heatmap for each sample. (C) Standardized (Z score) relative translation rates for the subset of proteins showing a decrease in translation correlating with global translation attenuation after titration of treatments. Clustering was performed on data from (B) and values of the most prominent cluster plotted for each treatment. Black lines indicate averaged curves from all displayed proteins. (D) Median relative translation rates of cells treated with indicated concentrations of 4EGI. (E) Heatmap displaying correlation of samples treated with different concentrations of either 4EGI, thapsigargin (Tg) or Torin1. Values represent Euclidean distance between samples. Clustering was performed over Euclidean distance. Apparent clusters are marked in red. (F) Heatmap displaying standardized relative translation values (Z score) for individual proteins following 4EGI treatment. (G) Standardized translation rates (Z score) for all proteins showing linear behavior of translation repression upon 4EGI titration (Figure S6B). See also Figure S6 and Table S5.

To validate the hypothesis that translation rates of most individual proteins correlate with global translation rates, we monitored dose-dependent translation attenuation using different concentrations of thapsigargin and Torin1 (Figure 6A). Clustering analysis showed that samples clustered based on global translation attenuation rate rather than on pathway (Figure 6B). We next analyzed the behavior of individual proteins after different treatment concentrations (Figure 6C). The biggest clusters of individual proteins followed a similar trend as the global translation (Figures 6A and 6C).

**Figure 7:**
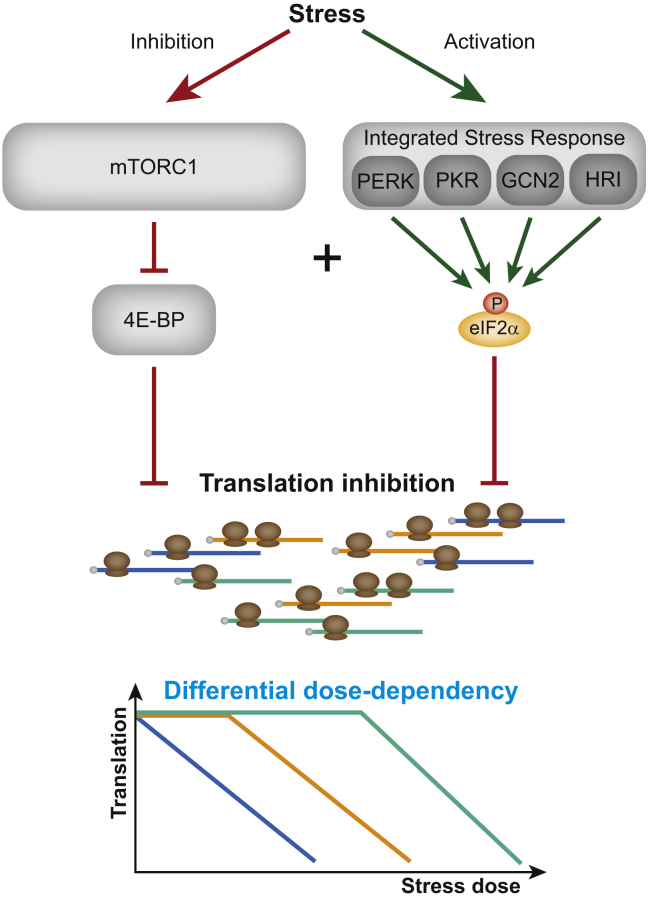
Model of Translation Regulation by mTORC1 and the Integrated Stress Response Model illustrating that the integrated stress response and mTORC1 regulate translation of an overlapping set of proteins despite their altering upstream regulation. Translation of individual proteins is largely explained by intrinsic factors with differential sensitivity of global translation inhibition as major determinant.

To evaluate this model on translation level without effects of the upstream pathways (i.e., ISR and mTORC1), we inhibited cap-dependent translation directly, using EIF4E/EIF4G interaction inhibitor 1 (4EGI) (Moerke et al., 2007). Titrating 4EGI caused dose-dependent translation attenuation (Figure 6D; Table S5). Comparing this data to translation inhibition with thapsigargin or Torin1 titration again showed clustering according to the grade of translation inhibition (Figure 6E), not inhibitor used, with three apparent major clusters representing different global translation rates. Cluster analysis on the level of individual proteins showed a major cluster correlating with global translation levels and with a near linear behavior of individual proteins (Figures 6F and S6A). Carrying out linear fits across all detected proteins revealed 2,190 proteins following this linear trend (Figure 6G and S6B), demonstrating that translation rates of the majority of cellular proteins directly correlate with global translation attenuation irrespective of the origin of translation attenuation (i.e., ISR or mTORC1). Notably, also these analyses exposed a fraction of proteins evading repression at all examined concentrations, consistent with previously published data describing core cellular pathways to be unaffected by inhibition of cap-dependent translation (Figure S6C) (Marques-Ramos et al., 2017). Together, these results demonstrate that individual protein translation upon stress is controlled by intrinsic factors, largely defining a threshold of global translation attenuation upon which translation of individual proteins ablates (Figure 7).

## Discussion

Determining the transcriptomes and proteomes of cells under various conditions has become a well-established standard used in many biological and medical applications. However, it has become clear that they correlate poorly and that monitoring the translatome as well is essential to understand protein synthesis and the regulation thereof (Ingolia et al., 2012, Maier et al., 2009). Ribo-seq has become the standard method to determine translation rates applied to many biological questions. However, it remains laborious, typically requires large amounts of sample material, and remains expensive (Ingolia et al., 2012, McGlincy and Ingolia, 2017). Thus, for many cellular conditions and stresses, particularly also in primary cells, translatome data are lacking, and its status and regulation is unknown, preventing understanding their role in cellular physiology. Furthermore, ribosome profiling can introduce a bias when measuring translation in states of global repression (Gandin et al., 2016, Masvidal et al., 2017), partially explaining critical differences in conclusions drawn from different experimental setups (Hsieh et al., 2012, Larsson et al., 2012, Morita et al., 2013, Thoreen et al., 2012).

To provide with a proteomics method complementary to Ribo-seq and to overcome some of its challenges, we developed mePROD that offers: (1) high sensitivity, allowing the measurement of highly acute differences in protein synthesis, (2) determination of translatome changes upon conditions with strong global translation shifts without normalization artifacts, and (3) an approach to quantify the translatome with limited sample input (i.e., around 100,000 cells) and at low cost (standard MS protocols and machines). Naturally, proteomic methods, including mePROD, do currently not provide with the same depth as Ribo-seq. Instead, mePROD offers direct information on nascent and newly synthesized proteins that present another layer of information directly related to translation. Due to its simplicity, sensitivity, and low price, mePROD may be applied to numerous biological questions not previously studied or applicable to Ribo-seq.

Key feature of mePROD is the inclusion of a “booster” channel that enables measurement of the signal of interest (newly synthesized peptides) by providing distinctive advantages: first, the booster channel only contains the signal of interest—heavy labeled peptides—thus specifically boosting the signal of newly translated proteins to reliably pass the limit of detection and identification. Second, the booster channel serves as an absolute reference point to allow determining translation relative to the booster channel and enables the comparison of samples analyzed in different LC-MS runs. In addition, mePROD also contains a noise channel comprised of light peptides to determine background noise levels and co-isolation interference for each individual peptide. This makes ratio compression, caused by co-isolation of non-targeted ions, as typically observed in TMT MS^2^-based methods, largely negligible. As a result, mePROD data acquisition can be carried out with MS^2^ methods, offering higher sensitivity and identification and quantification rates (Figures S1A and S1B). Together, mePROD enables translation proteomics with a temporal resolution capable of examining short-term changes of relative translation rates, as seen during stress responses.

mePROD offers various advantages for global translation quantification: (1) direct quantification of nascent chains, not relying on indirect sequence information, and (2) low input requirements in the range of typical proteomics experiments (< 100,000 cells) without the need of ribosome purification. Thus, mePROD is especially suitable for setups with limited starting material, such as clinical samples or primary cells. (3) No normalization bias, allowing ready quantification of individual and global protein translation rates, even in situations with global translation defects. At the same time, there are also method-inherent disadvantages driven by the use of mass spectrometry as a readout including an imperfect coverage or lack of detectability of proteins (due to sequence, abundance, and physical properties of peptides) and its limited depth, when compared to NGS based methods, where coverage mainly is a scalable function of sequencing depth. In addition, mePROD does not provide information on ribosome occupancy.

Despite eIF2α and EIF4EBP1—and thus the ISR and mTORC1—affecting processes in cap-dependent translation, it was generally assumed that the translational targets of eIF2α and EIF4EBP1 differ (Wengrod and Gardner, 2015). This is largely due to previous ribosome profiling analyses only uncovering small subsets of mRNAs with decreased translation that showed only minimal overlap between ISR and mTORC1 targets (Hsieh et al., 2012, Sidrauski et al., 2015, Thoreen et al., 2012). However, mePROD revealed the full extent of the extensive remodeling of the translational landscape upon stress induction (Figures 3 and 4). Comparison with previous datasets showed that mePROD identified most translationally regulated proteins revealed by ribosome profiling (Figures 5 and S3). It also detected the remodeling of translation in greater depth, resembling the global changes seen by ^35^S-Met metabolic labeling. By analyzing the detailed sets of translationally repressed proteins, we found that both pathways—ISR and mTORC1—have converging sets of targets (Figure 5). Crosstalk between both pathways is emerging as an interesting concept (Nikonorova et al., 2018, Zhang et al., 2018) in recent years, pointing to a complex picture of stress responses driving translational and transcriptional control. Nevertheless, the vast majority of translational changes cannot be explained by transcriptional patterns, since previous RNA-sequencing experiments did not show major effects when compared to our data (Figures S5D–S5G) (Andreev et al., 2015, Paolini et al., 2018, Thoreen et al., 2012).

We found the set of repressed target proteins to be determined by the strength of global translation repression rather than by the upstream pathway activated (Figures 4 and 6). In agreement with this hypothesis, titrating either the stress-inducing agents or a cap-dependent translation inhibitor showed dose-dependent effects on translation for the majority of proteins (Figure 6). Thus, features of each individual messenger RNA may reflect their sensitivity to translational changes (Figure 7). This model explained the vast majority of changes in the translatome upon modulating global translation. Strikingly, there is a small fraction of proteins not following this pattern, likely controlled by alternative translation initiation, or the specific transcriptional changes brought about by the ISR or mTORC1. These proteins include clusters of core cellular functions to retain their translation upon stress induction (Figures 3J, 4E, S4, and S6C). Consistent with previously published data (Marques-Ramos et al., 2017), we also found core signaling pathways to be maintained, most prominently the phosphate-inositol pathway and the mTOR pathway (Figure S6C). This might play a major role in cellular response to stresses that will result in a shut-down of protein translation of various subsets of proteins, dependent on the extent of stress (i.e., global translation attenuation), while keeping core pathways intact to ensure survival and function of cells during and after recovery from stress.

## STAR★Methods

### Key Resources Table

**Table:** 

REAGENT or RESOURCE	SOURCE	IDENTIFIER
**Antibodies**		

ACTB	SantaCruz	Cat#sc-69879; RRID:AB_1119529
EIF4EBP1	Cell Signaling Technologies	Cat#9644; RRID:AB_2097841
EIF2S1	Abcam	**Cat#ab5369; RRID:****AB_304838**
p-EIF2S1(S51)	Abcam	Cat#ab32157; RRID:AB_732117
IRDye 680RD anti-mouse	Li-Cor	Cat# 925-68070; RRID:AB_2651128
IRDye 800CW anti-rabbit	Li-Cor	Cat# 925-32211; RRID:AB_2651127

**Chemicals, Peptides, and Recombinant Proteins**		

Thapsigargin	Abcam	Cat#ab120286
2-Chloracetamide	Sigma Aldrich	Cat#C0267
Arginine 10	Cambridge Isotope Laboratories	Cat#CNLM-539-H-PK
Lysine 8	Cambridge Isotope Laboratories	Cat#CNLM-291-H-PK
**Sodium (meta)arsenite**	Sigma Aldrich	Cat#S7400
ISRIB	Sigma Aldrich	Cat#SML0843
4EGI-1	Selleckchem	Cat#S7369
TMT reagents	Thermo Fisher Scientific	Cat#90111, Cat#A37724, Cat#90061
Torin1	Cell Signaling Technologies	Cat#14379

**Critical Commercial Assays**		

μBCA microplate assay	Thermo Fisher Scientific	Cat#23235

**Deposited Data**		

RAW and quantified data	This paper/PRIDE	PXD014377 and PXD015438
Ribosome profiling and RNaseq data	Thoreen et al., 2012, Reid et al., 2014, Paolini et al., 2018, Andreev et al., 2015	https://doi.org/10.1038/nature11083, https://doi.org/10.1016/j.cell.2014.08.012, https://doi.org/10.1371/journal.pone.0193790, https://doi.org/10.7554/eLife.0397
SwissProt Human protein database	SwissProt	https://www.uniprot.org/downloads

**Experimental Models: Cell Lines**		

Human: HeLa		N/A

**Software and Algorithms**		

Python 3.6	Pyhton Consortium	https://www.python.org/; RRID:SCR_008394
pandas 0.23.4	McKinney, 2010	https://scipy.org/
Proteome Discoverer 2.2	ThermoFisher Scientific	Cat#OPTON-30795; RRID:SCR_014477
MaxQuant 1.6	Cox and Mann, 2008	https://www.maxquant.org/; RRID:SCR_014485
Perseus 1.6.2.3	Tyanova et al., 2016	https://www.maxquant.org/; RRID:SCR_015753
Numpy 1.15.4	van der Walt et al., 2011	https://scipy.org/; RRID:SCR_008633
Cytoscape 3.5.1	Shannon et al., 2003	https://cytoscape.org/; RRID:SCR_003032
BiNGO 3.0.3	Maere et al., 2005	https://www.psb.ugent.be/cbd/papers/BiNGO/Home.html; RRID:SCR_005736
EnrichmentMap 3.1.0	Merico et al., 2010	http://apps.cytoscape.org/apps/enrichmentmap; RRID:SCR_016052
Origin Pro 2018	OriginLab	https://www.originlab.com/2018; RRID:SCR_014212
ReactomeFI 6.1.0	Wu and Haw, 2017	http://apps.cytoscape.org/apps/reactomefiplugin
matplotlib 3.0.1	Hunter, 2007	https://scipy.org/; RRID:SCR_008624
scikit-learn 0.20.1	Pedregosa et al., 2011	https://scikit-learn.org/stable/index.html; RRID:SCR_002577

**Other**		

QExactive HF Orbitrap MS	Thermo Fisher Scientific	Cat#IQLAAEGAAPFALGMBFZ
Orbitrap Fusion Lumos Tribrid MS	Thermo Fisher Scientific	Cat#IQLAAEGAAPFADBMBHQ

### Lead Contact and Materials Availability

Further information and requests for resources and reagents should be directed to and will be fulfilled by the Lead Contact, Christian Münch (ch.muench@em.uni-frankfurt.de).

### Experimental Model and Subject Details

#### Cell lines and culture conditions

HeLa (human epithelial cervix-adenocarcinoma, female) cells were cultured in a humidified growth chamber at 37°C with 5% CO_2_ with RPMI1640 medium (GIBCO, 21875034) containing 10% FBS (GIBCO, 10270-106). To obtain fully labeled samples, cells were shifted to RPMI1640 medium for SILAC (GIBCO, 88365) containing 100 μg/mL Arg10 (Cambridge Isotope Laboratories), 100 μg/mL Lys8 (Cambridge Isotope Laboratories), 10% FBS and cultured for two weeks to ensure full label incorporation until cells were harvested.

For pulse labeling experiments, cells were untreated or treated for 30 min before pulse labeling (unless stated otherwise) with the desired compound (1 μM Thapsigargin [Abcam, ab120286]; 400 mM NaCl [Sigma Aldrich]; 0.5 mM Arsenite [Sigma Aldrich]; 500 nM ISRIB [Sigma Aldrich, SML0843]; 1 μM Torin1 [CST, 14379]; 4EGI [Selleckchem, S7369]) before washing two times with pre-warmed PBS (GIBCO) and incubation with SILAC medium containing the same concentration of the compound (where applicable) as the normal medium. Cells were grown in SILAC medium for an additional two hours (unless stated otherwise) until harvest.

### Method Details

#### Cell harvest and lysis

After labeling, cells were washed three times with warm PBS and lysed on the plate with lysis buffer (2% SDS, 50 mM Tris-HCl pH8, 150 mM NaCl, 10 mM TCEP, 40 mM chloracetamide, protease inhibitor cocktail tablet [EDTA-free, Roche] and Easy-phos phosphatase inhibitor tablet [Roche]). Lysates were scraped and transferred to 2 mL ProteinLoBind Eppendorf tubes (Eppendorf, Z666505). Samples were incubated for 5 min at 95°C before sonication with Sonic Vibra Cell at 1 s ON/ 1 s OFF pulse for 30 s at a maximal amplitude of 30% to shear genomic DNA. After sonication, samples were incubated for 10 min at 95°C.

#### Sample preparation for LC-MS^2^

Lysates were precipitated using three volumes of ice-cold methanol, one volume chloroform and 2.5 volumes ddH_2_O. After centrifugation at 14,000 g for 45 min at 4°C, the upper aqueous phase was aspirated and three volumes of ice-cold methanol added. Samples were mixed and proteins pelleted by centrifugation at 14,000 g for 5 min at 4°C. Supernatant was discarded and pellets washed one additional time with ice-cold methanol. Protein pellets were dried at room temperature for further use. Proteins were resuspended in 8 M Urea, 10 mM EPPS pH8.2, and 1 mM CaCl_2_ and protein concentration determined using a μBCA assay (ThermoFisher Scientific, 23235). Samples were then diluted to 2 M urea using digestion buffer (10 mM EPPS pH8.2, 1 mM CaCl_2_) and incubated with LysC (Wako Chemicals) at 1:50 (w/w) ratio overnight at 37°C. The next day digestion reactions were further diluted to 1 M Urea using digestion buffer and incubated at a 1:100 (w/w) ratio of Trypsin (Promega, V5113) for an additional 6 h at 37°C. Digests were acidified using trifluoroaceticacid (TFA) to a pH of 2-3 and peptides purified using SepPak C18 columns (Waters, WAT054955) according to the manufacturer’s protocol. Eluates were dried and stored for further processing.

Peptides were resuspended in TMT-labeling buffer (0.2 M EPPS pH8.2, 10% Acetonitrile) and peptide concentration determined by μBCA. Peptides were mixed with TMT reagents (ThermoFisher Scientific, 90111, A37724, 90061) in 1:2 (w/w) ratio (2 μg TMT reagent per 1 μg peptide). Reactions were incubated for one hour at RT and subsequently quenched by addition of hydroxylamine to a final concentration of 0.5% at RT for 15 min. Samples were pooled in equimolar ratio (unless stated otherwise), acidified, and dried for further processing.

Before MS-analysis, peptide samples were purified using Empore C18 (Octadecyl) resin material (3M Empore). Material was activated by incubation with Methanol for 5 min, followed by one wash each with 70% acetonitrile/0.1% TFA and 5% acetonitrile/0.1% TFA. Samples were resuspended in 5% acetonitrile, 0.1% TFA and loaded to resin material. Peptides were washed with 5% acetonitrile/0.1% TFA and eluted with 70% acetonitrile (ACN). Samples were dried and resuspended in 0.1% formic acid (FA) for LC-MS^2/3^.

#### High-pH Reverse Phase fractionation

Peptides were either fractionated using a Dionex Ultimate 3000 analytical HPLC or a High pH Reversed phase fractionation kit (ThermoFisher Scientific). The latter was used according to manufacturer’s instructions.

For high pH reversed phase fractionation on the Dionex HPLC, 500 μg of pooled and purified TMT-labeled samples were resuspended in 10 mM ammonium-bicarbonate (ABC), 5% ACN, and separated on a 250 mm long C18 column (Aeris Peptide XB-C18, 4.6 mm ID, 2.6 μm particle size; Phenomenex) using a multistep gradient from 100% Solvent A (5% ACN, 10 mM ABC in water) to 60% Solvent B (90% ACN, 10 mM ABC in water) over 70 min. Eluting peptides were collected every 45 s into a total of 96 fractions, which were cross-concatenated into 24 fractions and dried for further processing.

#### Mass spectrometry

Unless stated otherwise, peptides were resuspended in 0.1% FA and separated on an Easy nLC 1200 (ThermoFisher Scientific) and a 22 cm long, 75 μm ID fused-silica column, which had been packed in house with 1.9 μm C18 particles (ReproSil-Pur, Dr. Maisch), and kept at 45°C using an integrated column oven (Sonation). Peptides were eluted by a non-linear gradient from 5%–38% acetonitrile over 120 min and directly sprayed into a QExactive HF mass spectrometer equipped with a nanoFlex ion source (ThermoFisher Scientific) at a spray voltage of 2.3 kV. Full scan MS spectra (350-1400 m/z) were acquired at a resolution of 120,000 at m/z 200, a maximum injection time of 100 ms and an AGC target value of 3 × 10^6^. Up to 20 most intense peptides per full scan were isolated using a 1 Th window and fragmented using higher energy collisional dissociation (normalized collision energy of 35). MS/MS spectra were acquired with a resolution of 45,000 at m/z 200, a maximum injection time of 80 ms and an AGC target value of 1 × 10^5^. Ions with charge states of 1 and > 6 as well as ions with unassigned charge states were not considered for fragmentation. Dynamic exclusion was set to 20 s to minimize repeated sequencing of already acquired precursors.

Unfractionated test samples were separated on an Easy nLC II (ThermoFisher Scientific) and a 15 cm long, 75 μm ID fused-silica column, which has been packed in house with 3 μm C18 particles (ReproSil-Pur, Dr. Maisch), and kept at 45°C using an integrated column oven (Sonation). Peptides were eluted by a non-linear gradient from 5%–35% acetonitrile over 125 min and directly sprayed into a LTQ Orbitrap Elite mass-spectrometer equipped with a nanoFlex ion source (ThermoFisher Scientific) at a spray voltage of 2.3 kV. Full scan MS spectra (350-1650 m/z) were acquired at a resolution of 120,000 at m/z 200, a maximum injection time of 100 ms and an AGC target value of 1 × 10^6^ charges. Up to 20 most intense peptides per full scan were isolated in the ion-trap using a 2 Th window and fragmented using higher energy collisional dissociation (normalized collision energy of 35). MS/MS spectra were acquired with a resolution of 60,000 at m/z 200, a maximum injection time of 200 ms and an AGC target value of 5 × 10^4^. Ions with charge states of one as well as ions with unassigned charge states were not considered for fragmentation. Dynamic exclusion was set to 60 s to minimize repeated sequencing of already acquired precursors.

For MS^2^ and MS^3^ comparison, samples were shot on a Fusion Lumos Mass Spectrometer (Thermo Fisher Scientific). Peptides were resuspended in 0.1% FA and separated on an Easy nLC 1200 (ThermoFisher Scientific) and a 22 cm long, 75 μm ID fused-silica column, which has been packed in house with 1.9 μm C18 particles (ReproSil-Pur, Dr. Maisch), and kept at 45°C using an integrated column oven (Sonation). Peptides were eluted by a non-linear gradient from 5%–38% acetonitrile over 120 min and directly sprayed into a Fusion Lumos mass spectrometer equipped with a nanoFlex ion source (ThermoFisher Scientific) at a spray voltage of 2.6 kV. Full scan MS spectra (350-1400 m/z) were acquired at a resolution of 120,000 at m/z 200, a maximum injection time of 100 ms and an AGC target value of 1 × 10^6^ charges. Up to 15 most intense peptides per full scan were isolated using a 1 Th window and fragmented using higher energy collisional dissociation (normalized collision energy of 38). MS^2^ spectra were acquired with a resolution of 50,000 at m/z 200, a maximum injection time of 110 ms and an AGC target value of 5 × 10^4^. Ions with charge states of 1 and > 6 as well as ions with unassigned charge states were not considered for fragmentation. Dynamic exclusion was set to 45 s to minimize repeated sequencing of already acquired precursors.

For MS^3^ measurements, MS^2^ scans were performed in the IonTrap (Turbo) with an isolation window of 0.4 Th, a maximum injection time of 120 ms and CID fragmented using a collision energy of 35% for 10 ms. SPS-MS^3^ was performed on the 10 most intense MS^2^ fragment ions with an isolation window of 0.7 Th (MS^1^) and 2 m/z (MS^2^). Ions were fragmented using HCD with a normalized collision energy of 60 and analyzed in the Orbitrap with a resolution setting of 50,000 at m/z 200, scan range of 100-1000 m/z, AGC target value of 1.5 x10^5^ and a maximum injection time of 150 ms.

#### Western Blotting

Protein samples were separated by SDS-PAGE under reducing conditions. Proteins were transferred to 0.45 μM nitrocellulose membranes and probed with primary antibodies. Primary antibodies were used in 5% BSA in PBS in stated dilution (ACTB [SantaCruz] 1:5,000, EIF4EBP1 total [CST] 1:50,000, p-EIF2S1 [S51 Abcam] 1:2,000) for one hour at room temperature. Secondary antibodies (IRDye 680RD Donkey anti-mouse [Li-Cor], IRDye 800CW Donkey anti-rabbit [Li-Cor]) were used in 1:20,000 dilution in PBS and incubated for 30 min in the dark. Membranes were washed and imaged using an Odyssey CLx imager (Li-Cor).

### Quantification and Statistical Analysis

#### Processing of raw files

Raw files were analyzed using Proteome Discoverer (PD) 2.2 software (ThermoFisher Scientific). Files were recalibrated using the *Homo sapiens* SwissProt database (TaxID:9606, version 2017-06-07) with methionine oxidation (+15.995) as dynamic modification and carbamidomethyl (Cys,+57.021464), TMT6 (N-terminal, +229.1629) and TMT6 (+229.1629) at lysines as fixed modifications. Spectra were selected using default settings and database searches performed using SequestHT node in PD. Database searches were performed against trypsin digested *Homo sapiens* SwissProt database and FASTA files of common contaminants (`contaminants.fasta` provided with MaxQuant) for quality control. Fixed modifications were set as TMT6 at the N terminus and carbamidomethyl at cysteine residues. As dynamic modifications TMT6 (K), TMT6+K8 (K, +237.177), Arg10 (R, +10.008) and methionine oxidation were set. After search, posterior error probabilities were calculated and PSMs filtered using Percolator using default settings. Consensus Workflow for reporter ion quantification was performed with default settings, except the minimal signal-to-noise ratio was set to 5. Results were then exported to Excel files for further processing.

For SILAC only samples, raw files were analyzed using MaxQuant 1.6 (Cox and Mann, 2008), with default settings using the *Homo sapiens* SwissProt database (TaxID:9606, version 2017-06-07).

#### Data Analysis and statistics

Excel files were used as input for a custom made in-house Python pipeline. Python 3.6 was used together with the following packages: pandas 0.23.4 (McKinney, 2010), numpy 1.15.4 (van der Walt et al., 2011), matplotlib 3.0.1 (Hunter, 2007). Excel files with peptide data were read in and each channel was normalized to the lowest channel based on total intensity. For each peptide sequence, all possible modification states containing a heavy label were extracted and the intensities for each channel were averaged between all modified peptides. Baseline subtraction was performed by subtracting the measured intensities for the non-SILAC-labeled sample from all other values. Negative intensities were treated as zero. For relative quantification, channel values were divided by the abundance in the booster channel. The heavy label incorporation at the protein level was calculated by taking the median of all peptide sequences belonging to one unique protein accession. These values were combined with the standard protein output of PD 2.2 to add annotation data to the master protein accessions.

Log2 fold changes were calculated by log2 transformation of the ratio between the mean of the replicates of treated samples versus the control samples. Significance was assessed by unpaired, two-sided Student’s t test. *P value*s were adjusted by Benjamini-Hochberg FDR correction. Adjusted *P value*s lower than 0.05 were considered as significant. N represents number of independent replicates. Error bars, unless stated otherwise, indicate the standard deviation of replicates. Unless stated otherwise significance was defined as adjusted *P value*s < 0.05. Adjusted *P value* and fold change cutoffs were applied as indicated. For clustering and enrichment analyses (see below) q value cutoffs of 0.001 were used for significance definition.

Plotting and fitting of data was performed with Origin Pro 2018. For linear regression *P value*s were calculated with Origin and raw *P value*s used for statistics.

#### Multidimensional scaling

MDS was performed with Python 3.6 with scikit-learn 0.20.1 (Pedregosa et al., 2011) and pandas 0.23.4. Samples were standardized by removing the mean and scaled to unit variance. Resulting Z scores were subjected to MDS analysis with default settings. Dimensions were plotted using Origin Pro 2018 software.

#### Hierarchical clustering

Hierarchical cluster analysis for all samples was performed using Perseus (Tyanova et al., 2016) software package (version 1.6.2.3) with default settings after centering and scaling of data (Z scores).

#### Network analysis

For network analysis, Cytoscape 3.5.1 (Shannon et al., 2003) software was used with BiNGO 3.0.3 (Maere et al., 2005) plugin for GO term analysis, EnrichmentMap 3.1.0 (Merico et al., 2010) and ReactomeFI 6.1.0 (Wu and Haw, 2017). For GO-term analyses, gene sets were extracted from data as indicated using fold change and significance cutoffs. Gene sets were analyzed using BiNGO plugin with default settings for overrepresentation with GO sets for biological processes. Enrichment files were loaded into EnrichmentMap plugin for filtering. Q value cutoff was set to 0.001 as default and edge similarity cutoff was adjusted to 0.6.

For analysis on individual protein level, gene sets were analyzed using ReactomeFI. Gene sets were then FI annotated, clustered and modules were analyzed for Reactome pathway enrichment with a q-value cutoff of 0.001. Clusters were then manually annotated using the most prominent enriched pathways.

### Data and Code Availability

The mass spectrometry proteomics data have been deposited to the ProteomeXchange Consortium via the PRIDE (Perez-Riverol et al., 2019) partner repository with the dataset identifiers PXD015438 and PXD014377.

## Supplemental Information

Document S1. Figures S1–S6

Table S1. mePROD Translation Values in Response to Thapsigargin, Related to Figure 3 Translation was measured after 2.5 h of either DMSO, thapsigargin, or thapsigargin+ISRIB treatment. Provided are UniProt Identifiers, gene symbols, and expression values for each replicate and statistics (e.g., fold changes [log2] and adjusted p values).

Table S2. mePROD Translation Values in Response to Osmotic and Oxidative Stress, Related to Figure 4 Translation was measured after 2.5 h of either Vehicle, NaCl, or arsenite treatment. Provided are UniProt Identifiers, gene symbols, expression values for each replicate and statistics (e.g., fold changes [log2] and adjusted p values).

Table S3. mePROD Translation Values in Response to Osmotic and Oxidative Stress in Co-treatment with ISRIB, Related to Figure 4 Translation was measured after 2.5 h of either Vehicle, NaCl, or arsenite treatment together with ISRIB. Provided are UniProt Identifiers, gene symbols, expression values for each replicate and statistics (e.g., fold changes [log2] and adjusted p values).

Table S4. mePROD Translation Values in Response to Thapsigargin and Torin1, Related to Figure 5 Translation was measured after 2.5 h of either DMSO or thapsigargin treatment and 9 h of either DMSO or Torin1 treatment. Provided are UniProt Identifiers, gene symbols, expression values for each replicate and statistics (e.g., fold changes [log2] and adjusted p values).

Table S5. mePROD Translation Values in Response to 4EGI, Related to Figure 6 Translation was measured after 2.5 h of either Vehicle or increasing concentrations of 4EGI. Provided are UniProt Identifiers, gene symbols, expression values for each replicate and statistics (e.g., fold changes [log2] and adjusted p values).

Document S2. Article plus Supplemental Information

## References

[bib1] Andreev D.E., O’Connor P.B., Fahey C., Kenny E.M., Terenin I.M., Dmitriev S.E., Cormican P., Morris D.W., Shatsky I.N., Baranov P.V. (2015). Translation of 5′ leaders is pervasive in genes resistant to eIF2 repression. eLife.

[bib2] Back S.H., Scheuner D., Han J., Song B., Ribick M., Wang J., Gildersleeve R.D., Pennathur S., Kaufman R.J. (2009). Translation attenuation through eIF2α phosphorylation prevents oxidative stress and maintains the differentiated state in β cells. Cell Metab..

[bib3] Becher I., Andrés-Pons A., Romanov N., Stein F., Schramm M., Baudin F., Helm D., Kurzawa N., Mateus A., Mackmull M.-T. (2018). Pervasive Protein Thermal Stability Variation during the Cell Cycle. Cell.

[bib4] Bevilacqua E., Wang X., Majumder M., Gaccioli F., Yuan C.L., Wang C., Zhu X., Jordan L.E., Scheuner D., Kaufman R.J. (2010). eIF2α phosphorylation tips the balance to apoptosis during osmotic stress. J. Biol. Chem..

[bib5] Chen K., Hu Z., Xia Z., Zhao D., Li W., Tyler J.K. (2015). The Overlooked Fact: Fundamental Need for Spike-In Control for Virtually All Genome-Wide Analyses. Mol. Cell. Biol..

[bib6] Clavarino G., Cláudio N., Couderc T., Dalet A., Judith D., Camosseto V., Schmidt E.K., Wenger T., Lecuit M., Gatti E., Pierre P. (2012). Induction of GADD34 is necessary for dsRNA-dependent interferon-β production and participates in the control of Chikungunya virus infection. PLoS Pathog..

[bib7] Colina R., Costa-Mattioli M., Dowling R.J.O., Jaramillo M., Tai L.-H., Breitbach C.J., Martineau Y., Larsson O., Rong L., Svitkin Y.V. (2008). Translational control of the innate immune response through IRF-7. Nature.

[bib8] Cox J., Mann M. (2008). MaxQuant enables high peptide identification rates, individualized p.p.b.-range mass accuracies and proteome-wide protein quantification. Nat. Biotechnol..

[bib9] De Benedetti A., Graff J.R. (2004). eIF-4E expression and its role in malignancies and metastases. Oncogene.

[bib10] Dowling R.J.O., Topisirovic I., Alain T., Bidinosti M., Fonseca B.D., Petroulakis E., Wang X., Larsson O., Selvaraj A., Liu Y. (2010). mTORC1-mediated cell proliferation, but not cell growth, controlled by the 4E-BPs. Science.

[bib11] DuRose J.B., Scheuner D., Kaufman R.J., Rothblum L.I., Niwa M. (2009). Phosphorylation of eukaryotic translation initiation factor 2α coordinates rRNA transcription and translation inhibition during endoplasmic reticulum stress. Mol. Cell. Biol..

[bib12] Gandin V., Masvidal L., Hulea L., Gravel S.-P., Cargnello M., McLaughlan S., Cai Y., Balanathan P., Morita M., Rajakumar A. (2016). nanoCAGE reveals 5′ UTR features that define specific modes of translation of functionally related MTOR-sensitive mRNAs. Genome Res..

[bib13] Graff J.R., Zimmer S.G. (2003). Translational control and metastatic progression: enhanced activity of the mRNA cap-binding protein eIF-4E selectively enhances translation of metastasis-related mRNAs. Clin. Exp. Metastasis.

[bib14] Harding H.P., Novoa I., Zhang Y., Zeng H., Wek R., Schapira M., Ron D. (2000). Regulated translation initiation controls stress-induced gene expression in mammalian cells. Mol. Cell.

[bib15] Holz M.K., Ballif B.A., Gygi S.P., Blenis J. (2005). mTOR and S6K1 mediate assembly of the translation preinitiation complex through dynamic protein interchange and ordered phosphorylation events. Cell.

[bib16] Hsieh A.C., Liu Y., Edlind M.P., Ingolia N.T., Janes M.R., Sher A., Shi E.Y., Stumpf C.R., Christensen C., Bonham M.J. (2012). The translational landscape of mTOR signalling steers cancer initiation and metastasis. Nature.

[bib17] Hunter J.D. (2007). Matplotlib: A 2D Graphics Environment. Comput. Sci. Eng..

[bib18] Ingolia N.T., Brar G.A., Rouskin S., McGeachy A.M., Weissman J.S. (2012). The ribosome profiling strategy for monitoring translation in vivo by deep sequencing of ribosome-protected mRNA fragments. Nat. Protoc..

[bib19] Jiang Z., Yang J., Dai A., Wang Y., Li W., Xie Z. (2017). Ribosome profiling reveals translational regulation of mammalian cells in response to hypoxic stress. BMC Genomics.

[bib20] Jovanovic M., Rooney M.S., Mertins P., Przybylski D., Chevrier N., Satija R., Rodriguez E.H., Fields A.P., Schwartz S., Raychowdhury R. (2015). Immunogenetics. Dynamic profiling of the protein life cycle in response to pathogens. Science.

[bib21] Kozak M. (1999). Initiation of translation in prokaryotes and eukaryotes. Gene.

[bib22] Krishnamoorthy T., Pavitt G.D., Zhang F., Dever T.E., Hinnebusch A.G. (2001). Tight binding of the phosphorylated α subunit of initiation factor 2 (eIF2α) to the regulatory subunits of guanine nucleotide exchange factor eIF2B is required for inhibition of translation initiation. Mol. Cell. Biol..

[bib23] Larsson O., Morita M., Topisirovic I., Alain T., Blouin M.-J., Pollak M., Sonenberg N. (2012). Distinct perturbation of the translatome by the antidiabetic drug metformin. Proc. Natl. Acad. Sci. U. S. A..

[bib24] Lee A.-H., Iwakoshi N.N., Glimcher L.H. (2003). XBP-1 regulates a subset of endoplasmic reticulum resident chaperone genes in the unfolded protein response. Mol. Cell. Biol..

[bib26] Maere S., Heymans K., Kuiper M. (2005). BiNGO: a Cytoscape plugin to assess overrepresentation of gene ontology categories in biological networks. Bioinformatics.

[bib27] Maier T., Güell M., Serrano L. (2009). Correlation of mRNA and protein in complex biological samples. FEBS Lett..

[bib28] Marques-Ramos A., Candeias M.M., Menezes J., Lacerda R., Willcocks M., Teixeira A., Locker N., Romão L. (2017). Cap-independent translation ensures mTOR expression and function upon protein synthesis inhibition. RNA.

[bib29] Masvidal L., Hulea L., Furic L., Topisirovic I., Larsson O. (2017). mTOR-sensitive translation: Cleared fog reveals more trees. RNA Biol..

[bib30] Mathieson T., Franken H., Kosinski J., Kurzawa N., Zinn N., Sweetman G., Poeckel D., Ratnu V.S., Schramm M., Becher I. (2018). Systematic analysis of protein turnover in primary cells. Nat. Commun..

[bib31] McEwen E., Kedersha N., Song B., Scheuner D., Gilks N., Han A., Chen J.-J., Anderson P., Kaufman R.J. (2005). Heme-regulated inhibitor kinase-mediated phosphorylation of eukaryotic translation initiation factor 2 inhibits translation, induces stress granule formation, and mediates survival upon arsenite exposure. J. Biol. Chem..

[bib32] McGlincy N.J., Ingolia N.T. (2017). Transcriptome-wide measurement of translation by ribosome profiling. Methods.

[bib33] McKinney W. (2010). Data Structures for Statistical Computing in Python. Proceedings of the 9th Python in Science Conference.

[bib34] Merico D., Isserlin R., Stueker O., Emili A., Bader G.D. (2010). Enrichment map: a network-based method for gene-set enrichment visualization and interpretation. PLoS ONE.

[bib35] Miura H., Hashida K., Sudo H., Awa Y., Takarada-Iemata M., Kokame K., Takahashi T., Matsumoto M., Kitao Y., Hori O. (2010). Deletion of Herp facilitates degradation of cytosolic proteins. Genes Cells.

[bib36] Moerke N.J., Aktas H., Chen H., Cantel S., Reibarkh M.Y., Fahmy A., Gross J.D., Degterev A., Yuan J., Chorev M. (2007). Small-molecule inhibition of the interaction between the translation initiation factors eIF4E and eIF4G. Cell.

[bib37] Morita M., Gravel S.-P., Chénard V., Sikström K., Zheng L., Alain T., Gandin V., Avizonis D., Arguello M., Zakaria C. (2013). mTORC1 controls mitochondrial activity and biogenesis through 4E-BP-dependent translational regulation. Cell Metab..

[bib38] Münch C., Harper J.W. (2016). Mitochondrial unfolded protein response controls matrix pre-RNA processing and translation. Nature.

[bib39] Nikonorova I.A., Mirek E.T., Signore C.C., Goudie M.P., Wek R.C., Anthony T.G. (2018). Time-resolved analysis of amino acid stress identifies eIF2 phosphorylation as necessary to inhibit mTORC1 activity in liver. J. Biol. Chem..

[bib40] Pakos‐Zebrucka K., Koryga I., Mnich K., Ljujic M., Samali A., Gorman A.M. (2016). The integrated stress response. EMBO Rep..

[bib41] Paolini N.A., Moore K.S., di Summa F.M., Fokkema I.F.A.C., ’t Hoen P.A.C., von Lindern M. (2018). Ribosome profiling uncovers selective mRNA translation associated with eIF2 phosphorylation in erythroid progenitors. PLoS ONE.

[bib42] Park Y., Reyna-Neyra A., Philippe L., Thoreen C.C. (2017). mTORC1 Balances Cellular Amino Acid Supply with Demand for Protein Synthesis through Post-transcriptional Control of ATF4. Cell Rep..

[bib43] Pedregosa F., Varoquaux G., Gramfort A., Michel V., Thirion B., Grisel O., Blondel M., Prettenhofer P., Weiss R., Dubourg V. (2011). Scikit-learn: Machine Learning in Python. J. Mach. Learn. Res..

[bib44] Perez-Riverol Y., Csordas A., Bai J., Bernal-Llinares M., Hewapathirana S., Kundu D.J., Inuganti A., Griss J., Mayer G., Eisenacher M. (2019). The PRIDE database and related tools and resources in 2019: improving support for quantification data. Nucleic Acids Res..

[bib45] Plescher M., Teleman A.A., Demetriades C. (2015). TSC2 mediates hyperosmotic stress-induced inactivation of mTORC1. Sci. Rep..

[bib46] Preston A.M., Hendershot L.M. (2013). Examination of a second node of translational control in the unfolded protein response. J. Cell Sci..

[bib47] Prostko C.R., Brostrom M.A., Brostrom C.O. (1993). Reversible phosphorylation of eukaryotic initiation factor 2 α in response to endoplasmic reticular signaling. Mol. Cell. Biochem..

[bib48] Rabouw H.H., Langereis M.A., Anand A.A., Visser L.J., de Groot R.J., Walter P., van Kuppeveld F.J.M. (2019). Small molecule ISRIB suppresses the integrated stress response within a defined window of activation. Proc. Natl. Acad. Sci. USA.

[bib49] Raught B., Peiretti F., Gingras A.-C., Livingstone M., Shahbazian D., Mayeur G.L., Polakiewicz R.D., Sonenberg N., Hershey J.W. (2004). Phosphorylation of eucaryotic translation initiation factor 4B Ser422 is modulated by S6 kinases. EMBO J..

[bib50] Reid D.W., Chen Q., Tay A.S.-L., Shenolikar S., Nicchitta C.V. (2014). The unfolded protein response triggers selective mRNA release from the endoplasmic reticulum. Cell.

[bib51] Ron D., Walter P. (2007). Signal integration in the endoplasmic reticulum unfolded protein response. Nat. Rev. Mol. Cell Biol..

[bib52] Roux P.P., Topisirovic I. (2012). Regulation of mRNA translation by signaling pathways. Cold Spring Harb. Perspect. Biol..

[bib53] Sabatini D.M. (2006). mTOR and cancer: insights into a complex relationship. Nat. Rev. Cancer.

[bib54] Savitski M.M., Zinn N., Faelth-Savitski M., Poeckel D., Gade S., Becher I., Muelbaier M., Wagner A.J., Strohmer K., Werner T. (2018). Multiplexed Proteome Dynamics Profiling Reveals Mechanisms Controlling Protein Homeostasis. Cell.

[bib55] Schwanhäusser B., Gossen M., Dittmar G., Selbach M. (2009). Global analysis of cellular protein translation by pulsed SILAC. Proteomics.

[bib56] Schwanhäusser B., Busse D., Li N., Dittmar G., Schuchhardt J., Wolf J., Chen W., Selbach M. (2011). Global quantification of mammalian gene expression control. Nature.

[bib57] Shannon P., Markiel A., Ozier O., Baliga N.S., Wang J.T., Ramage D., Amin N., Schwikowski B., Ideker T. (2003). Cytoscape: a software environment for integrated models of biomolecular interaction networks. Genome Res..

[bib58] Sidrauski C., McGeachy A.M., Ingolia N.T., Walter P. (2015). The small molecule ISRIB reverses the effects of eIF2α phosphorylation on translation and stress granule assembly. eLife.

[bib59] Sonenberg N., Hinnebusch A.G. (2009). Regulation of translation initiation in eukaryotes: mechanisms and biological targets. Cell.

[bib60] Taniuchi S., Miyake M., Tsugawa K., Oyadomari M., Oyadomari S. (2016). Integrated stress response of vertebrates is regulated by four eIF2α kinases. Sci. Rep..

[bib61] Thoreen C.C., Chantranupong L., Keys H.R., Wang T., Gray N.S., Sabatini D.M.M. (2012). A unifying model for mTORC1-mediated regulation of mRNA translation. Nature.

[bib62] Tyanova S., Temu T., Sinitcyn P., Carlson A., Hein M.Y., Geiger T., Mann M., Cox J. (2016). The Perseus computational platform for comprehensive analysis of (prote)omics data. Nat. Methods.

[bib63] van der Walt S., Colbert S.C., Varoquaux G. (2011). The NumPy Array: A Structure for Efficient Numerical Computation. Comput. Sci. Eng..

[bib64] Wang X., Li W., Williams M., Terada N., Alessi D.R., Proud C.G. (2001). Regulation of elongation factor 2 kinase by p90(RSK1) and p70 S6 kinase. EMBO J..

[bib65] Welle K.A., Zhang T., Hryhorenko J.R., Shen S., Qu J., Ghaemmaghami S. (2016). Time-resolved Analysis of Proteome Dynamics by Tandem Mass Tags and Stable Isotope Labeling in Cell Culture (TMT-SILAC) Hyperplexing. Mol. Cell. Proteomics.

[bib66] Wengrod J.C., Gardner L.B. (2015). Cellular adaptation to nutrient deprivation: crosstalk between the mTORC1 and eIF2α signaling pathways and implications for autophagy. Cell Cycle.

[bib67] Wu G., Haw R. (2017). Functional Interaction Network Construction and Analysis for Disease Discovery.

[bib68] Yoshida H., Matsui T., Yamamoto A., Okada T., Mori K. (2001). XBP1 mRNA is induced by ATF6 and spliced by IRE1 in response to ER stress to produce a highly active transcription factor. Cell.

[bib69] Zhang S., Macias-Garcia A., Velazquez J., Paltrinieri E., Kaufman R.J., Chen J.-J. (2018). HRI coordinates translation by eIF2αP and mTORC1 to mitigate ineffective erythropoiesis in mice during iron deficiency. Blood.

