# Supplementary material for: Functional Translatome Proteomics Reveal Converging and Dose-Dependent Regulation by mTORC1 and eIF2α
Source: Mol Cell. 2020 Feb 20;77(4):913–925.e4. doi: 10.1016/j.molcel.2019.11.010 (PMC7033560; doi:10.1016/j.molcel.2019.11.010)
Supplement: Document S1. Figures S1–S6 [file mmc1.pdf]

Molecular Cell, Volume 77

Supplemental Information

Functional Translatome Proteomics Reveal Converging and Dose-Dependent Regulation by mTORC1 and eIF2α

Kevin Klann, Georg Tascher, and Christian Münch

SUPPLEMENTARY FIGURE LEGENDS

Figure S1. mePROD accurately determines acute changes in relative protein translation rates, Related to Figure 1

(A-B) Ratio compression of mePROD measurements. Comparison of samples from Figure 2 measured using MS^2 or MS^3 methods (A) and with or without noise subtraction employing the noise channel (B).

(C) Depiction of median measured relative translation rates (heavy/total) across time points (Figure 2H) relative to the determined dynamic range of mePROD (Figure 2E).

(D) Reproducibility of translation values for individual protein across different time points (Figure 2H). For each time-point, measured translation values were scaled between 0 and 1 and individual scaled translation values as well as calculated curve fits were plotted.

Figure S2. Assessment of quality of mePROD measurements, Related to Figure 3

(A) Heatmap showing correlation between all replicates. Pearson correlation matrix was calculated and plotted. Tg, thapsigargin.

(B) Distribution of fold changes between DMSO or thapsigargin treated samples.

(C) Distribution of adjusted *P* values between DMSO or thapsigargin treated samples. Raw *P* values were calculated using two-sided, unpaired student's t-test (n=3) and FDR-corrected by the Benjamini Hochberg procedure.

Figure S3. Validation translation targets by comparison to existing ribosome profiling studies, Related to Figure 3

(A) Comparison of Ribo-seq and mePROD translation data after activation of the integrated stress response. Plotted are the fractions of proteins overlapping between the Ribo-seq (indicated publications as below) and mePROD data sets (Figure 3).

(B, C) Histograms of relative translation rate fold changes observed by mePROD or Ribo-seq data from (Reid et al., 2014) (B), or (Paolini et al., 2018) (C).

Figure S4. Effect of endoplasmic reticulum stress on the functional translatome, Related to Figure 3/4

(A) Venn diagram showing the overlap between proteins with reduced translation in thapsigargin versus DMSO or thapsigargin+ISRIB treated cells (fold change (log2) < -0.5).

(B) Relative GO term associations for the whole quantified translatome dataset compared to proteins with decreased translation (fold change < -0.5) upon thapsigargin treatment.

(C) Volcano plot showing translation rate fold changes of thapsigargin (Tg) and ISRIB versus DMSO treated cells and statistical significance. Raw *P* values were calculated using two-sided,

unpaired student’s t-test (n=3) and FDR-corrected by the Benjamini Hochberg procedure. Significantly changing proteins are marked in orange.

(D) GO term network analysis of translatome changes upon thapsigargin treatment showing proteins with unchanged (orange) or decreased (blue, fold change < -0.5) translation.
(E) Scheme of Mannose-6-phosphate receptor (MPR) trafficking in cells and the effect of thapsigargin on the pathway. Shown are thapsigargin/control fold changes (log2) for the main pathway components.

Figure S5. Overlap of proteins with reduced translation after folding-, oxidative-, or osmotic stress, Related to Figure 4
(A) Venn-diagram showing overlap between proteins showing significant translation decrease (fold change < -0.5, adjusted *P* value < 0.05) upon treatment with thapsigargin, NaCl, or arsenite. Only proteins detected in all datasets are shown.
(B) Western blot showing eiF2α (total EIF2S1) and P-eIF2α (P-EIF2S1) levels upon control, NaCl or arsenite treatments for 2.5 h with or without ISRIB co-treatment.
(C) Venn diagram showing proteins whose translation attenuation after arsenite (Ars), NaCl, or thapsigargin (Tg) treatment could be rescued by addition of ISRIB (Fold change > 0.5).
(D) Transcriptional fold changes extracted from previously published datasets following UPR induction (Paolini et al., 2018), arsenite treatment (Andreev et al., 2015), or pharmacological mTOR inhibition (Thoreen et al., 2012) were plotted. Median fold changes are indicated on top.
(E–G) Transcriptional fold changes from datasets used in (D) plotted against mePROD translation data (Figure 4). Pearson correlation values are indicated within the figures. Ctrl, control. FC, fold change.

Figure S6.Translation behavior of different protein clusters during 4EGI titration, Related to Figure 6
(A) Cluster profiles of the two main clusters identified from Figure 6F. Z scores were plotted for each sample. Greyscale indicates distance from the cluster center. Dashed line indicates linear reference line.
(B) Translation rates of all proteins present in dataset were normalized to control sample and linear fitting carried out. All individual proteins (n=2,190) following the linear fit with a *P* value lower than 0.05 were plotted. The dashed line represents the averaged curve.
(C) Reactome FI network of proteins retaining their translational status over all tested 4EGI concentrations.

Figure S1

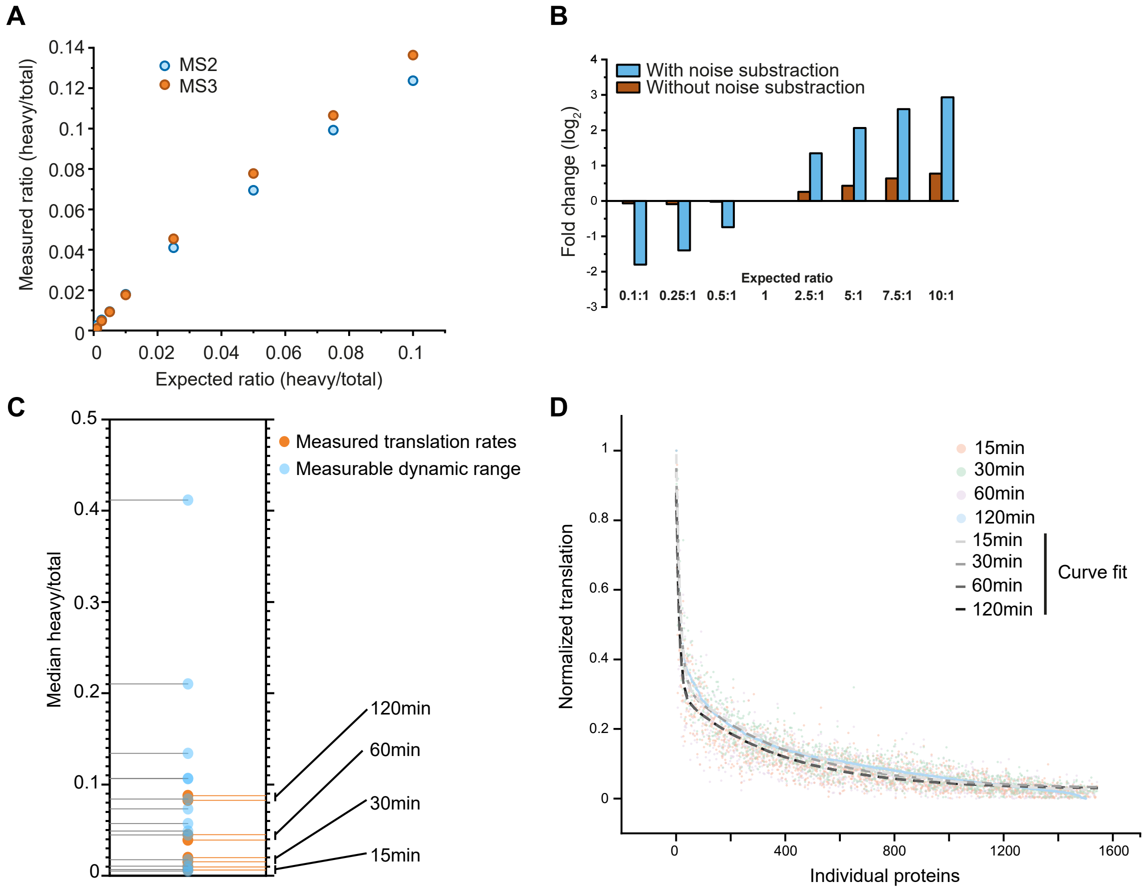
A
MS2
MS3
Measured ratio (heavy/total)
Expected ratio (heavy/total)
0.14
0.12
0.1
0.08
0.06
0.04
0.02
0
B
With noise substraction
Without noise substraction
Fold change (log2)
Expected ratio
0.1:1
0.25:1
0.5:1
1
2.5:1
5:1
7.5:1
10:1
C
Measured translation rates
Measurable dynamic range
Median heavy/total
120min
60min
30min
15min
D
Normalized translation
Individual proteins
15min
30min
60min
120min
Curve fit

Figure S2

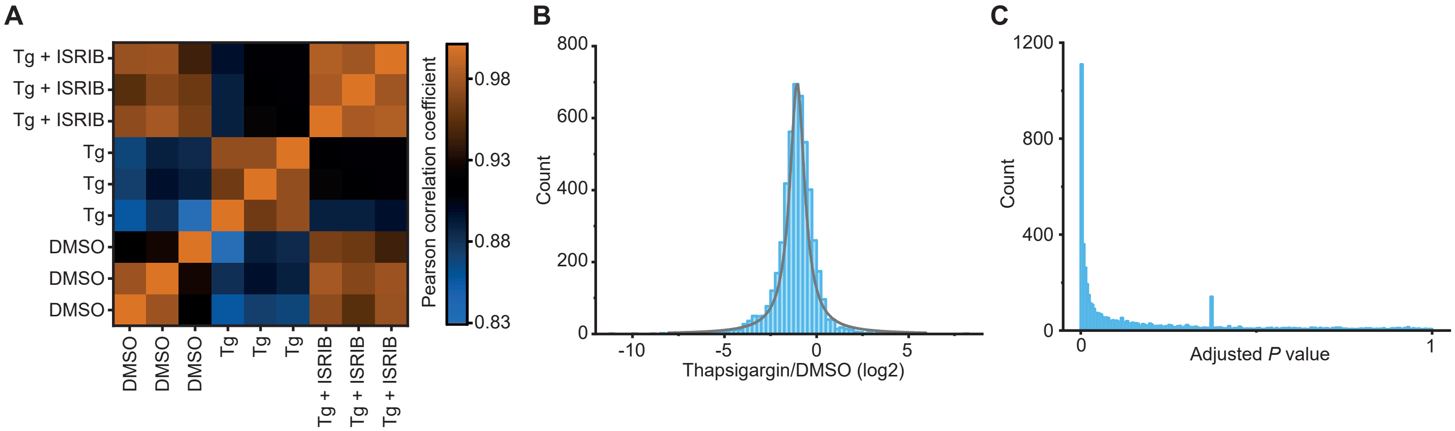
A
Tg + ISRIB
Tg + ISRIB
Tg + ISRIB
Tg
Tg
Tg
DMSO
DMSO
DMSO
DMSO
DMSO
DMSO
Tg
Tg
Tg
Tg + ISRIB
Tg + ISRIB
Tg + ISRIB
Pearson correlation coefficient
0.98
0.93
0.88
0.83
B
800
600
400
200
0
Count
-10
-5
0
5
Thapsigargin/DMSO (log2)
C
1200
800
400
0
Count
0
1
Adjusted P value

Figure S3

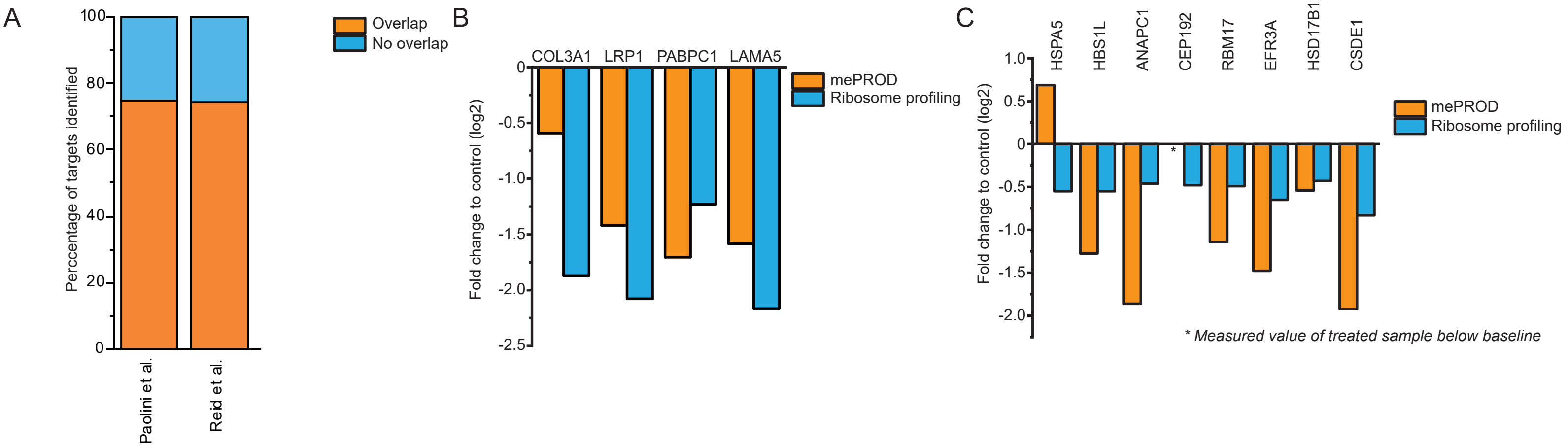
A
100
80
60
40
20
0
Percentage of targets identified
Overlap
No overlap
Paolini et al.
Reid et al.
B
COL3A1
LRP1
PABPC1
LAMA5
0
-0.5
-1.0
-1.5
-2.0
-2.5
Fold change to control (log2)
mePROD
Ribosome profiling
C
HSPA5
HBS1L
ANAPC1
CEP192
RBM17
EFR3A
HSD17B12
CSDE1
1.0
0.5
0
-0.5
-1.0
-1.5
-2.0
Fold change to control (log2)
mePROD
Ribosome profiling
*
* Measured value of treated sample below baseline

Figure S4

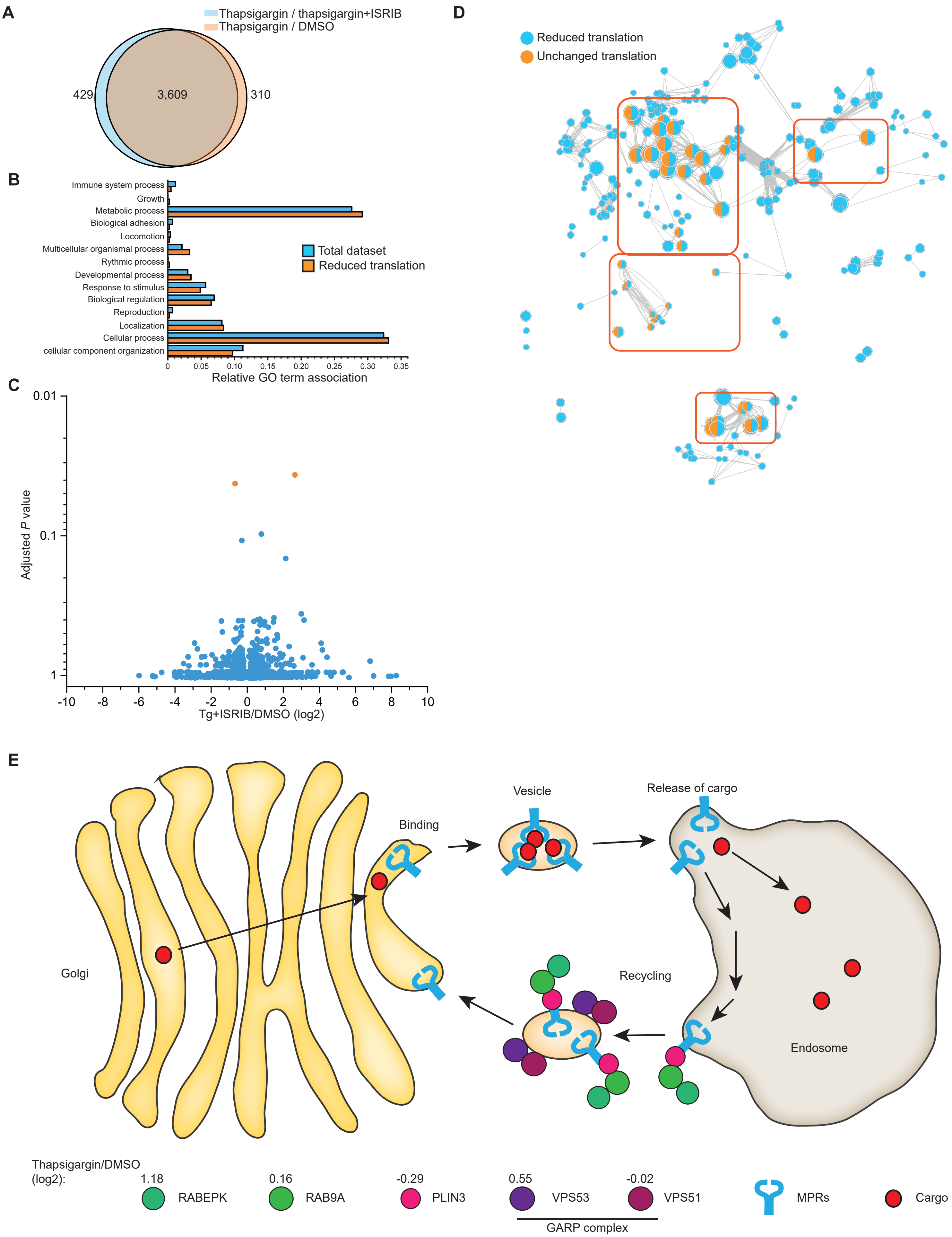

A
Thapsigargin / thapsigargin+ISRIB
Thapsigargin / DMSO
429
3,609
310
B
Immune system process
Growth
Metabolic process
Biological adhesion
Locomotion
Multicellular organismal process
Rythmic process
Developmental process
Response to stimulus
Biological regulation
Reproduction
Localization
Cellular process
cellular component organization
Total dataset
Reduced translation
0 0.05 0.10 0.15 0.20 0.25 0.30 0.35
Relative GO term association
C
0.01
0.1
1
Adjusted P value
-10 -8 -6 -4 -2 0 2 4 6 8 10
Tg+ISRIB/DMSO (log2)
D
Reduced translation
Unchanged translation
E
Golgi
Binding
Vesicle
Release of cargo
Recycling
Endosome
Thapsigargin/DMSO (log2):
1.18
RABEPK
0.16
RAB9A
-0.29
PLIN3
0.55
VPS53
-0.02
VPS51
GARP complex
MPRs
Cargo

Figure S5

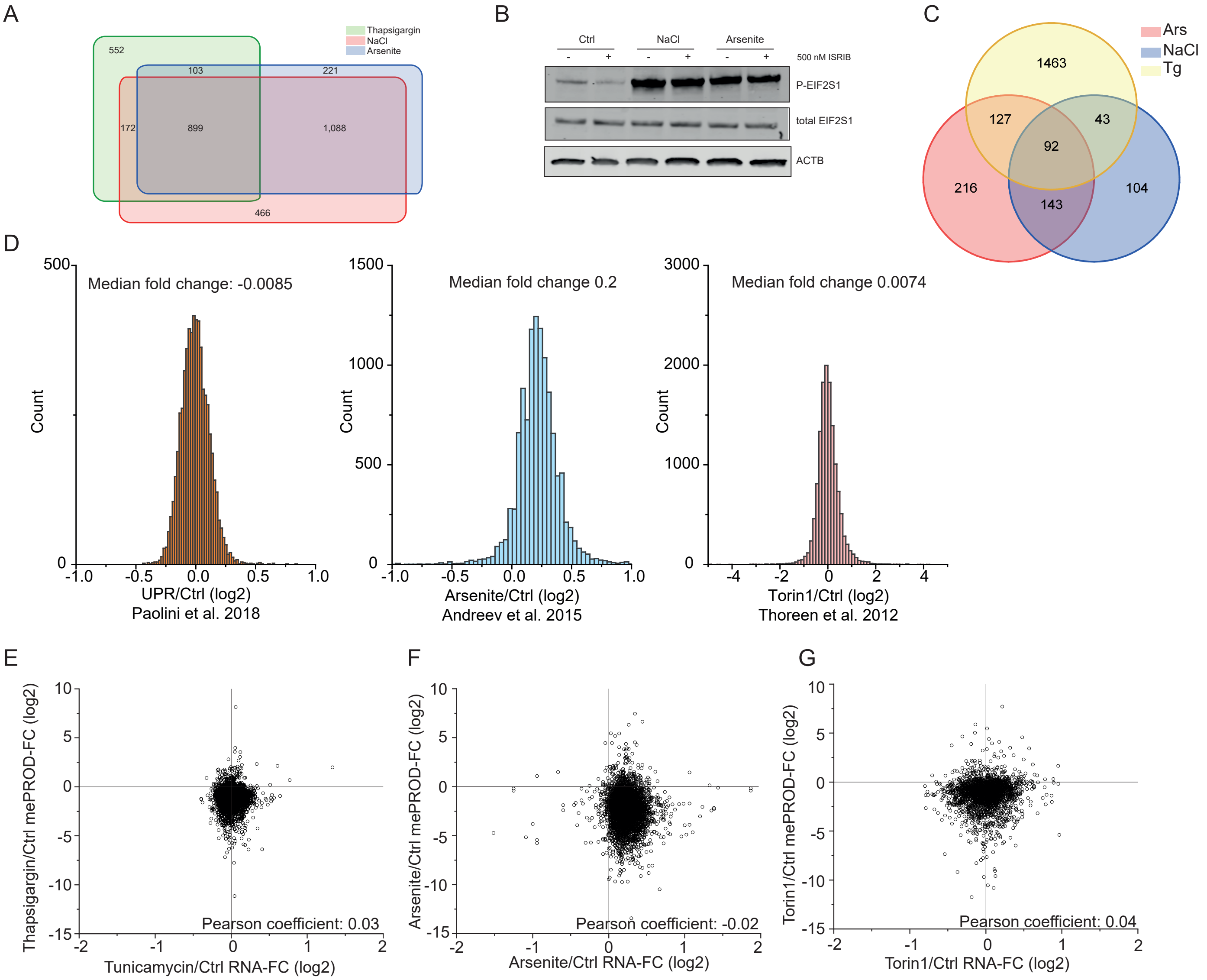
A
Thapsigargin
NaCl
Arsenite
552
103
221
172
899
1,088
466
B
Ctrl
NaCl
Arsenite
500 nM ISRIB
- + - + - +
P-EIF2S1
total EIF2S1
ACTB
C
Ars
NaCl
Tg
1463
127
43
92
216
143
104
D
Median fold change: -0.0085
Count
UPR/Ctrl (log2)
Paolini et al. 2018
Median fold change 0.2
Count
Arsenite/Ctrl (log2)
Andreev et al. 2015
Median fold change 0.0074
Count
Torin1/Ctrl (log2)
Thoreen et al. 2012
E
Thapsigargin/Ctrl mePROD-FC (log2)
Pearson coefficient: 0.03
Tunicamycin/Ctrl RNA-FC (log2)
F
Arsenite/Ctrl mePROD-FC (log2)
Pearson coefficient: -0.02
Arsenite/Ctrl RNA-FC (log2)
G
Torin1/Ctrl mePROD-FC (log2)
Pearson coefficient: 0.04
Torin1/Ctrl RNA-FC (log2)

Figure S6

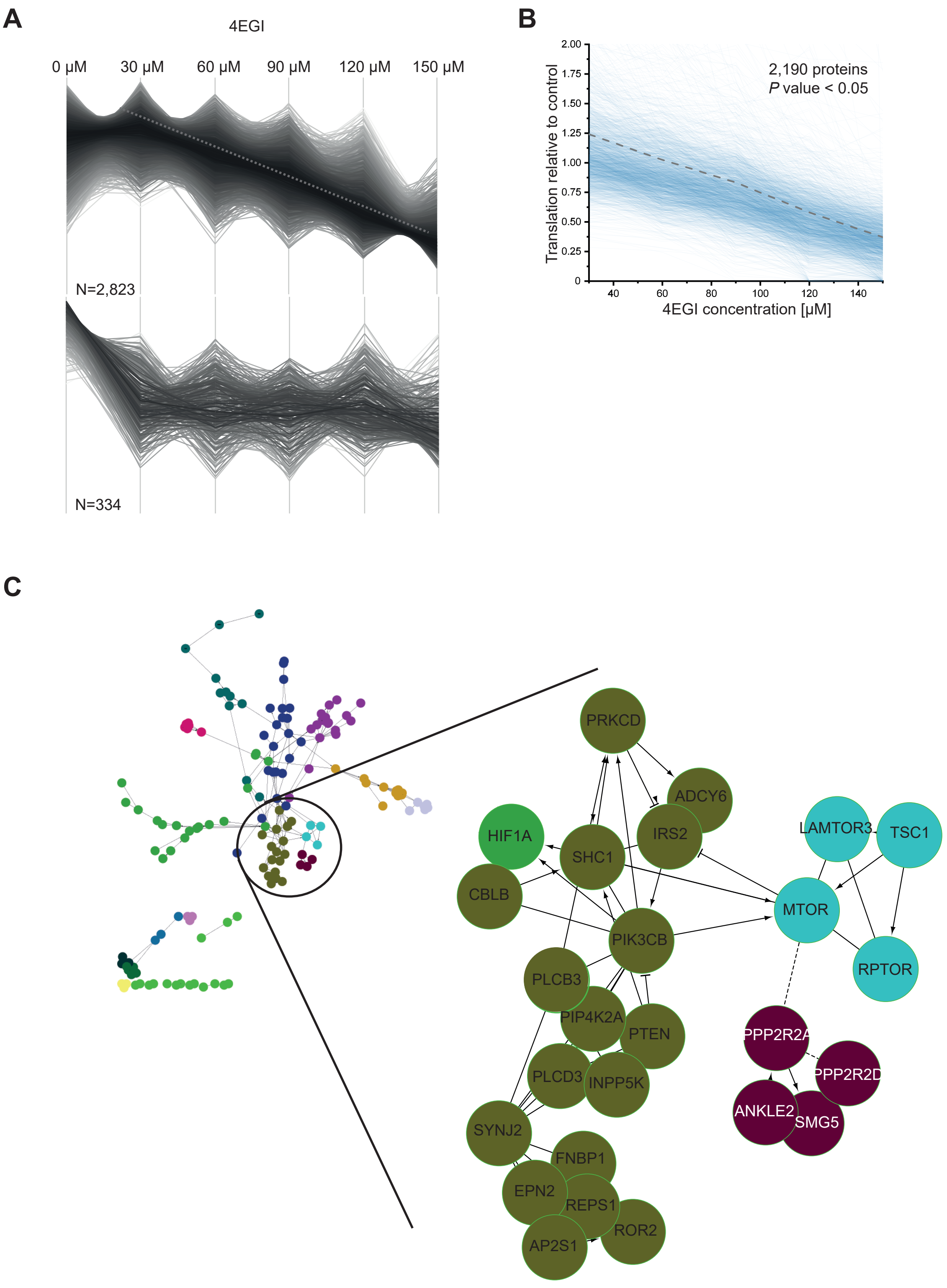

A
4EGI
0 μM
30 μM
60 μM
90 μM
120 μM
150 μM
N=2,823
N=334
B
2,190 proteins
P value < 0.05
Translation relative to control
2.00
1.75
1.50
1.25
1.00
0.75
0.50
0.25
0
40
60
80
100
120
140
4EGI concentration [μM]
C
PRKCD
ADCY6
HIF1A
SHC1
IRS2
LAMTOR3
TSC1
CBLB
MTOR
PIK3CB
RPTOR
PLCB3
PIP4K2A
PTEN
PPP2R2A
PPP2R2D
PLCD3
INPP5K
ANKLE2
SMG5
SYNJ2
FNBP1
EPN2
REPS1
ROR2
AP2S1
